# Electrical control of hybrid exciton transport in a van der Waals heterostructure

**DOI:** 10.1038/s41566-023-01198-w

**Published:** 2023-04-20

**Authors:** Fedele Tagarelli, Edoardo Lopriore, Daniel Erkensten, Raül Perea-Causín, Samuel Brem, Joakim Hagel, Zhe Sun, Gabriele Pasquale, Kenji Watanabe, Takashi Taniguchi, Ermin Malic, Andras Kis

**Affiliations:** 1 Institute of Electrical and Microengineering, École Polytechnique Fédérale de Lausanne (EPFL), Lausanne, Switzerland; 2 Institute of Materials Science and Engineering, École Polytechnique Fédérale de Lausanne (EPFL), Lausanne, Switzerland; 3 Department of Physics, Chalmers University of Technology, Gothenburg, Sweden; 4 Department of Physics, Philipps-Universität Marburg, Marburg, Germany; 5 Research Center for Functional Materials, National Institute for Materials Science, Tsukuba, Japan; 6 International Center for Materials Nanoarchitectonics, National Institute for Materials Science, Tsukuba, Japan

**Keywords:** Nanoscale devices, Nanoscale materials

## Abstract

Interactions between out-of-plane dipoles in bosonic gases enable the long-range propagation of excitons. The lack of direct control over collective dipolar properties has so far limited the degrees of tunability and the microscopic understanding of exciton transport. In this work we modulate the layer hybridization and interplay between many-body interactions of excitons in a van der Waals heterostructure with an applied vertical electric field. By performing spatiotemporally resolved measurements supported by microscopic theory, we uncover the dipole-dependent properties and transport of excitons with different degrees of hybridization. Moreover, we find constant emission quantum yields of the transporting species as a function of excitation power with radiative decay mechanisms dominating over nonradiative ones, a fundamental requirement for efficient excitonic devices. Our findings provide a complete picture of the many-body effects in the transport of dilute exciton gases, and have crucial implications for studying emerging states of matter such as Bose–Einstein condensation and optoelectronic applications based on exciton propagation.

## Abstract (web summary)

The dipole-dependent propagation of hybrid excitons in a van der Waals heterostructure containing a WSe_2_ bilayer is characterized by modulating the layer hybridization and interplay between many-body interactions of excitons with an applied vertical electric field.

## Main

Exciton transport has been proposed as a potential basis for realizing scaled optical interconnects and modulators in chip-scale optical processing systems^1^. Strongly bound and long-lived propagating excitons can act as information carriers within a semiconductor—a desirable prospect for photonic circuits^2,3^. In particular, spatially indirect excitons can propagate with micrometre-scale diffusion lengths. They can be controlled via the quantum-confined Stark effect, enabling tuning of their potential energy by an applied vertical electric field^4,5^. Van der Waals heterostructures of two-dimensional (2D) materials have been used as platforms for manipulating spatially indirect interlayer excitons (IXs)^6^. In particular, type-II transition metal dichalcogenide (TMDC) heterostructures have been employed to realize excitonic devices^7^ and circuits^8^. Heterostructure devices showing room-temperature switching of exciton currents^7^, tunable valley-polarized emission^9^ and micrometre-scale transport of polarized exciton currents^10^ have been demonstrated.

The spatial separation of charges comprising IXs gives rise to fixed out-of-plane dipole moments^11,12^. Repulsive Coulomb interactions between exciton populations of out-of-plane dipolar ensembles induce anomalous transport dynamics that deviate from the standard diffusive propagation that is characteristic of bosonic gases^13–16^. Instead, excitons generated in single TMDC layers, also called intralayer excitons, are mainly influenced by quantum-mechanical exchange interactions^17^. The interplay between dipolar and exchange interactions strongly depends on the out-of-plane exciton dipole length. Layer-hybridized states with intra- and interlayer components are therefore expected to show variable effective out-of-plane dipole lengths^18^. The ability to control the degree of exciton hybridization is highly desirable as a means to modify the concurrent many-body interactions and tune the anomalous diffusion of exciton ensembles. While layer-hybridized states in twisted moiré heterostructures and their Stark shifts with applied electric fields have been previously investigated^19–21^, such type-II heterostructures do not allow tuning of exciton–exciton interactions because the dipole moment of the emitting species is intrinsically fixed by layer arrangement. Moreover, the moiré superlattice that is formed in stacked bilayers has been shown to induce periodic potential traps that dramatically reduce the effective diffusivity of out-of-plane excitons^22,23^. This effect hampers reliable long-range exciton transport, making moiré-less structures preferable for manipulating long-range propagating dipolar gases in excitonic devices.

Here we exploit concurrent intervalley transitions in natural WSe_2_ homobilayers to control the layer hybridization of exciton states by applying a vertical electric field. 2H-WSe_2_ homobilayers are moiré-less structures that have been indicated to be a natural platform for Bose–Einstein condensation of IX states^24^. However, an in-depth study of the dynamics and transport of layer-hybridized tunable exciton states in this platform is still lacking. Furthermore, we achieve electrostatic control over hybrid IX (hIX) transport in a structure with no moiré potential by varying the interplay between Coulombic dipolar repulsions and attractive exchange interactions. Our work sheds light on the influence of dipole length and hybridization on long-range IX transport and is supported by a microscopic theory. Moreover, we show that the propagating exciton species in this platform are characterized by a quantum yield that is constant in power with dominant radiative recombination channels, independently of the layer hybridization. The study of electrically tunable dipolar ensembles with constant quantum yield and micrometre-scale transport opens the way to realizing efficient excitonic devices based on van der Waals heterostructures of 2D materials.

## Results

### Electrically tunable interlayer dipolar ensembles

**Fig. 1:**
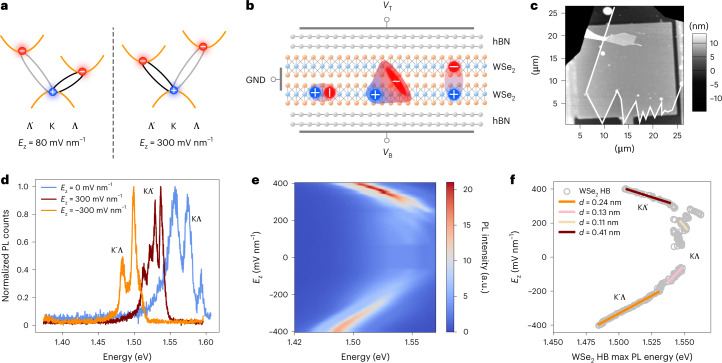
Electrically tunable interlayer dipolar ensembles in a van der Waals homobilayer. **a**, Schematic band structure of a natural WSe_2_ homobilayer that hosts different dominant intervalley transitions depending on *E*_*z*_ (Supplementary Note 2). With a positive *E*_*z*_, the main favourable transition shifts from KΛ to KΛ′, with increasing interlayer mixing and sizeable out-of-plane dipole moments. **b**, A double-gated fully hBN-encapsulated natural homobilayer WSe_2_ device with graphical representations of intralayer (left), hybrid (centre) and purely interlayer (right) exciton species. *V*_T_ and *V*_B_ indicate the applied top and bottom gate voltages, respectively, and GND indicates ground. **c**, Atomic force microscopy image of device A, with a large clean area (>80 μm^2^) that exhibits uniform height (greyscale bar) and excitonic properties (Supplementary Note 1). The WSe_2_ homobilayer edge is highlighted by a white solid line. **d**, PL spectra acquired for different electric fields in device A, featuring the emission from hIX phonon replicas. The labels indicate dominant exciton transitions. **e**, PL spectra as a function of the applied vertical electric field. Low (|*E*_*z*_| < 200 mV nm^−1^) and high (|*E*_*z*_| ≥ 200 mV nm^−1^) field regions are related to predominant KΛ/K′Λ′ and KΛ′/K′Λ transitions, respectively. **f**, Extracted energy of the highest intensity PL peak from the PL spectra as a function of *E*_*z*_ in the WSe_2_ homobilayer (HB). The energy shift as a function of the electric field is fitted to a line in low- and high-field regimes, revealing variable dipole moments. In particular, larger dipole lengths (*d* > 0.2 nm) are related to high-field regions dominated by KΛ′ and K′Λ transitions, which are characterized by high interlayer mixing (Supplementary Note 2) .

Our devices consisted of natural WSe_2_ homobilayers, fully encapsulated in hexagonal boron nitride (hBN), with a bottom Cr/Pt gate and a top semi-transparent Pt gate. The heterostacks were assembled on an SiO_2_ substrate by mechanical transfer (Methods). Figure 1c shows the image of an ultra-clean encapsulated bilayer WSe_2_ device (A), acquired by a.c.-mode atomic force microscopy. Images of a second device (B) are included in Supplementary Note 1. Natural 2H-WSe_2_ homobilayers host momentum-indirect spin-bright KΛ and KΛ′ transitions as the energetically lowest states^25,26^ involving holes at the K/K′ points and electrons at the Λ/Λ′ points of the Brillouin zone (Fig. 1a). Given their indirect nature, excited states in WSe_2_ appear in the photoluminescence (PL) spectrum as their phonon replicas^25^. Intervalley excitons in bilayer WSe_2_ are hybrid^27^ in their intra- and interlayer components^28^. Our device architecture (Fig. 1b) allowed us to modulate the PL emission from layer-hybridized intervalley excitons in bilayer WSe_2_ with an increasing applied vertical electric field *E*_*z*_, causing a shift in the lowest-state transition from KΛ (K′Λ′) to KΛ′ (K′Λ) (Supplementary Table 1 and Supplementary Note 2). The states in brackets represent degenerate states with opposite dipole moments^18^. In the presence of a positive vertical field with magnitude *E*_*z*_ = 300 mV nm^−1^, the interlayer mixing coefficient of the energetically lowest state (KΛ′) was calculated to be $\left| {C_{\mathrm{IX}}^{{\mathrm{K}}{{\Lambda }}^\prime }} \right|^2 = 0.80$. We note that hybrid excitons are expected to have higher oscillator strengths than purely interlayer species, as they exhibit higher radiative decay rates. This is a desirable feature for higher-temperature operation. However, further studies are needed to evaluate the influence of nonradiative dynamics on hIX recombination at room temperature. In this work, we focused on the performance and tunability of hIX transport at a temperature of 4 K.

The potential energy *U* of out-of-plane electron–hole pairs with fixed dipole lengths *d* can be modulated by the linear quantum-confined Stark effect as $\updelta U \approx dE_z$. The degree of interlayer character of hIXs is highly tunable via the application of a vertical electric field. It can then be observed through the Stark effect acting on the out-of-plane component of the transitions of interest. From the field-dependent PL spectra of device A (Fig. 1d,e), we could distinguish two main ranges corresponding to the favourable transitions KΛ and K′Λ′ ($\left| {E_z} \right| < 200\,{{{\mathrm{mV}}}}\,{{{\mathrm{nm}}}}^{ - 1}$) or KΛ′ and K′Λ ($\left| {E_z} \right| > 200\,{{{\mathrm{mV}}}}\,{{{\mathrm{nm}}}}^{ - 1}$). The peaks assigned to the corresponding transitions are discussed in Supplementary Note 2. From the linear Stark shift of the PL peaks with the highest intensity, we extracted different effective out-of-plane dipole lengths *d*_eff_ with respect to the vertical field on the basis of the prevalent emitting states^29^ (Fig. 1f and Supplementary Note 3). The brightest PL peak can shift between different phonon replicas. In particular, we observed a clear shift from the low-field to the high-field dominant transition at $\left| {E_z} \right| = 200\,{{{\mathrm{mV}}}}\,{{{\mathrm{nm}}}}^{-1}$. The electric field ranges corresponding to specific dominant transitions are highlighted in Fig. 1f. In particular, KΛ and K′Λ′ excitons were characterized by smaller dipole lengths ($d_{{{{\mathrm{eff}}}}} \simeq 0.1\,{{{\mathrm{nm}}}}$) with respect to the KΛ′ and K′Λ counterparts (*d*_eff_ > 0.2 nm)^29,30^. In our case, high positive and high negative vertical electric fields linked to the KΛ′ and K′Λ transitions are related to dipolar ensembles 0.41 nm and 0.24 nm long, respectively (Supplementary Note 3). Asymmetries in the field-dependent behaviour of out-of-plane transitions have been reported as a function of doping^31^. In Supplementary Note 4 we show how the collective dipole moment of high-*d* transitions can be effectively modulated by gating. In fact, effective out-of-plane dipole lengths of collective ensembles can be tuned by induced or intrinsic doping^31^. In particular, *d*_eff_ modulations on the order of ångströms are attributed to the electric field screening of the exciton wavefunctions. Thus, we attributed the difference between the two branches in the Stark shift measurements of Fig. 1e to the presence of intrinsic doping in the WSe_2_ homobilayers used in this work.

### Field-effect control of hybrid exciton transport

**Fig. 2:**
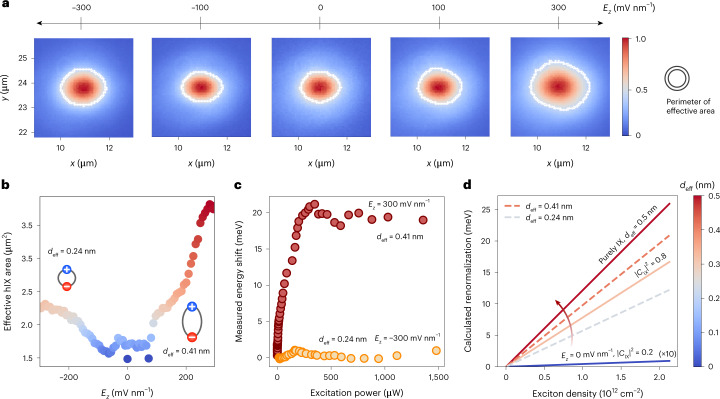
Field-effect control of hybrid exciton transport. **a**, Spatial images of steady-state hIX PL cloud expansion taken by a CCD camera (Methods) as a function of the applied vertical electric field. The perimeter of the effective area is defined as the 1/*e* points of the normalized PL intensity, and is shown as a white contour in all images for display purposes. **b**, Steady-state effective hIX cloud area plotted with respect to *E*_*z*_. With increasing *E*_*z*_, sizeable collective out-of-plane dipoles result in an increase in the repulsive interactions driving hIX diffusion and enhanced steady-state transport. The blue and red extremes correspond to negligible and high dipole lengths (right colour scale). The maximum areas obtained at positive and negative fields are related by a factor of ~1.7, equivalent to the *d*_hd_/*d*_ld_ ratio between the corresponding dipole lengths of 0.41 nm (*d*_hd_) and 0.24 nm (*d*_ld_), respectively. **c**, Extracted peak energy shift of the PL emission from the KΛ′ and K′Λ transitions at high positive and negative electric fields as a function of the incident laser excitation power. Both species are characterized by linear blueshifts for *P*_in_ < 150 μW, followed by a saturation (the origin of which is discussed in detail in Supplementary Note 6). **d**, Theoretical calculation of the energy normalization shift in an ideal WSe_2_ homobilayer as a function of *E*_*z*_. Higher *E*_*z*_ induces stronger interlayer mixing in the layer hybridization, with different linear energy shift tendencies and effective dipole lengths. A multiplication factor (x10) was applied to the data for negligible *E*_*z*_ (blue line).

Our system hosts layer-hybridized excitons characterized by different effective lengths, allowing us to determine the dipole-dependent transport properties of strongly interacting exciton gases in the dilute regime. Purely IX gases with large separations between electrons and holes are characterized by negligible exchange forces^14,17,32^. Hybrid ensembles, however, host sizeable intra- and interlayer components at every given field^33^. Thus, both Coulombic dipolar repulsions and attractive exchange interactions give rise to renormalized hybrid exciton energies. Their interplay is dictated by the level of interlayer hybridization, which we modulated by applying a vertical electric field. By tuning the hybridization and *d*_eff_ of the probed excitons, we achieved control over the concurrent many-body interactions in the micrometre-scale transport of dilute exciton gases. We studied the tunable many-body interactions by measuring the steady-state effective diffusion area of hIXs in the presence of an applied electric field (Fig. 2a). With negligible *E*_*z*_, short-range exciton transport was observed due to the prevalence of the intralayer component in the energetically degenerate KΛ and K′Λ′ states. Such low-field transitions featured randomly oriented dipoles and negligible repulsive interactions. However, with higher positive or negative fields, sizeable out-of-plane dipole lengths and greater interlayer mixing of hybrid states resulted in stronger collective repulsive forces. Consequently, we were able to electrostatically enhance the steady-state exciton gas expansion by a progressive transition from low-*d* to high-*d* dominated ensembles with increasing interlayer components (Fig. 2b).

We could extract a lower-bound estimate of purely IX densities from the measured blueshift using the parallel-plate capacitor model^34^. However, layer-hybridized excitons in WSe_2_ homobilayers show sizeable attractive exchange interactions, resulting in a density-dependent redshift that counteracts the effect of dipolar repulsive Coulomb forces. To delve into the many-body picture of strongly interacting dipolar gases, we developed a microscopic theory that accounts for the two main components driving excitonic transport in hybrid forms. We studied hIX interactions by deriving a hybrid exciton–exciton interaction Hamiltonian that we used to disentangle the main contributions to the density-dependent exciton energy renormalization (Supplementary Note 5):

1 $${{\Delta }}E^\xi \left( {n_x} \right) = n_x^{\bar {\xi} }g_{\mathrm{d - d}}^{\xi \bar {\xi} } + n_x^\xi \left( {g_{\mathrm{d - d}}^{\xi \xi } + g_{x - x}^{\xi \xi }} \right)$$

where $g_{\mathrm{d - d}}$ is the dipole–dipole interaction strength, which is negligible for intralayer excitons in monolayer TMDCs and dominates for spatially separated IXs; $g_{x - x}$ accounts for exchange interactions, which are highly dependent on the out-of-plane separation for interlayer states^33^. The interactions are weighted by the valley-specific exciton density $n_x^\xi$, where the total exciton density *n*_*x*_ is given by $n_x = \mathop {\sum}\nolimits_\xi {n_x^\xi }$. Equation (1) takes into account all contributions from the different intervalley transitions, with $\xi = {\mathrm{K}}{{\Lambda }},{\mathrm{K}}^\prime {{\Lambda }}^\prime ,{\mathrm{K}}{{\Lambda }}^\prime ,{\mathrm{K}}^\prime {{\Lambda }}$ (${\bar {\xi}}$ denotes the opposite valley, that is if $\xi = {\mathrm{K}}{{\Lambda }}$, then ${\bar {\xi}} = {\mathrm{K}}^\prime {{\Lambda }}^\prime$). In Fig. 2c,d, we show that the density-dependent energy normalization for hIXs in WSe_2_ homobilayers is highly dependent on the vertical electric field as the hybrid exciton–exciton interaction crucially depends on the interlayer mixing coefficients. We have calculated the energy shifts for layer-hybridized excitons in undoped WSe_2_ homobilayers by solving a hybrid exciton eigenvalue problem that accounts for mixing between intra- and interlayer exciton states^18^ (Supplementary Note 2).

The *d*_eff_ of the hIXs was extracted by fitting a linear function to the energy shift $\Delta E^\xi = n_xd_{{{{\mathrm{eff}}}}}^\xi /{\it{\epsilon }}$, with ${\it{\epsilon }} = {\it{\epsilon }}_0{\it{\epsilon }}_ \bot$ where ${\it{\epsilon }}_0$ is the vacuum permittivity and ${\it{\epsilon }}_ \bot$ is the out-of-plane component of the dielectric tensor of the TMDC. The tunable effective out-of-plane dipole length of the exciton species is directly related to the level of layer hybridization, with *d*_IX_ = 0.5 nm for purely interlayer states. For the simulated ideal energy shifts for the dominant KΛ′ transition (interlayer mixing $\left| {C_{\mathrm{IX}}^{{\mathrm{K}}{{\Lambda }}^\prime }} \right|^2 = 0.8$ for *E*_*z*_ = 300 mV nm^−1^), we predicted *d*_eff_ = 0.32 nm. The calculated value is consistent with our measured values ranging from 0.24 nm to 0.41 nm in the presence of intrinsic doping. While excitons with a negligible interlayer component show no density-dependent shift in energy, as discussed in Supplementary Note 5, species with sizeable net out-of-plane dipole lengths show an increase in the magnitude of the blueshift at higher vertical electric fields. We characterized the measured renormalization shifts from high-*d* (0.41 nm) and low-*d* (0.24 nm) ensembles by observing a linear blueshift at low optical excitation intensity (*P*_in_ < 150 μW) followed by Δ*E* saturation. We ascribed this saturation to lattice heating at high exciton densities, as previously observed with spatially indirect excitons in double quantum well systems (Supplementary Note 6).

### Radiative recombination of hIXs with a power-independent quantum yield

**Fig. 3:**
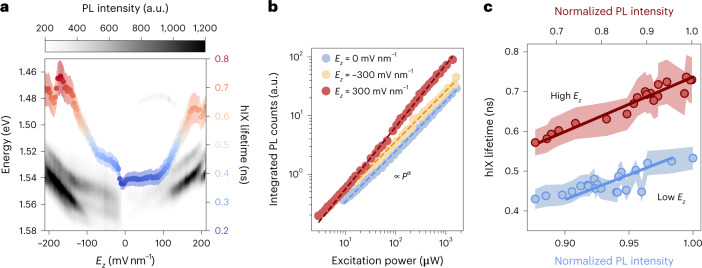
Radiatively recombining hIXs with power-independent quantum yield. **a**, The measured hIX lifetime in device B (colour) superimposed on the PL spectral map (greyscale) as a function of the applied vertical electric field. The lifetime is extracted from a single exponential fit to time-resolved PL measurements (Supplementary Note 7). Lifetime data are presented as mean values (dots) and corresponding standard deviations (shaded envelope). The hIX lifetime increases with respect to the applied vertical electric field due to a smaller electron–hole wavefunction overlap at higher interlayer hybridizations. **b**, Integrated PL counts recorded at high positive, negative and zero electric fields in device A as a function of laser excitation power. A constant emission quantum yield is recorded independently of *E*_*z*_ and of the layer hybridization level. The extended power regime in which PL counts are linear with respect to excitation power indicates that nonradiative decay mechanisms are negligible in the probed hIX dynamics. Shaded lines represent linear fits to the respective data points, giving *I*_hIX_ ∝ *P*^α^ for all curves, with 0.9 ≤ α ≤ 1. **c**, A linear relationship is found between the hIX lifetime *t*_hIX_ at high (−180 mV nm^−1^ < *E*_*z*_ < −110 mV nm^−1^, in red) and low (−100 mV nm^−1^ < *E*_*z*_ < −25 mV nm^−1^, in blue) electric fields and their PL intensity *I*_hIX_, extracted from the data shown in **a**. The shaded areas represent the standard deviation of the lifetime data from **a**. Blue and red solid lines represent linear fits to the respective data points. The *t*_hIX_ ∝ *I*_hIX_ trend in our structure is independent of the layer hybridization and is explained by the predominance over radiative recombination channels in the transporting species (Supplementary Note 7).

Spatially separated exciton species are characterized by longer radiative lifetimes with respect to their intralayer counterparts as their electron and hole wavefunctions feature a smaller overlap and, consequently, a lower probability of recombining^13^. Figure 3a shows the Stark shift of the main PL peaks and their measured lifetime in device B, highlighting the relationship between field-dependent lifetime and the change in the lowest-state emitting intervalley species. Comparable results were obtained in device A, as reported in Supplementary Note 7. As a result of the longer effective out-of-plane dipole length of the hybrid excitations with higher fields, we observed increased lifetimes when the main emitting transition shifted from KΛ (K′Λ′) to KΛ′ (K′Λ). Excitons belonging to the former states in device B were characterized by average lifetimes of 0.40 ns, whereas the maximum value reached for the latter was around 0.75 ns (device A in Supplementary Note 7). The hBN thicknesses in device B were low enough to enable photo-assisted tunnelling of carriers at high electric fields (*E*_*z*_ > 200 mV nm^−1^) in the form of a photocurrent (Supplementary Note 7). We attribute the sudden drop in the hIX lifetime at large positive and negative fields to the dissociation of excitons and their tunnelling through the hBN barriers to the top and bottom gate electrodes^35,36^ (Supplementary Note 7).

It has been shown that the main nonradiative channel that affects the quantum yield of TMDCs is power-dependent exciton–exciton annihilation^37^. In our case, we observed a single-exponential time-resolved PL decay independently of the applied vertical electric field and of the input optical power (Supplementary Note 7). Moreover, a linear relationship between the integrated PL intensity and the input pump intensity indicated that the quantum yield of the probed exciton species was constant with power (Fig. 3b). On the basis of these findings, we concluded that hIXs in WSe_2_ homobilayers do not recombine via second-order power-dependent nonradiative channels. This is a significant difference from IXs in previously investigated type-II heterostructures. For purely IXs in type-II structures, density-dependent nonradiative terms have been shown to induce a decrease in the emission quantum yield of the dipolar ensembles at high excitation intensities^10,13,14^. We note that, consistently with our findings, excitons in WSe_2_ homobilayers have been reported to feature suppressed exciton–exciton annihilation channels at room temperature, with a power-independent quantum yield that does not require chemical treatment or induced strain^38^. This can be explained by the decreased overlap between excitonic wavefunctions due to hybridization^39^, together with the absence of higher-lying states that fulfil energy and momentum conservation^40^.

However, even though a single power-independent decay channel is found for our hIXs, further analysis is required to determine its radiative and nonradiative composition. Jauregui et al.^13^ have established that it is possible to retrieve quantitative information about nonradiative decay channels from the field-dependent lifetime of purely IXs. A nonradiative decay rate was found even at low power for purely interlayer species in MoSe_2_/WSe_2_. By applying the same approach to the field-dependent lifetime of hIXs, we observed a linear increase in the maximum PL intensity with respect to lifetime (Fig. 3c). A linear trend with a positive coefficient, together with the constant quantum yield and single-exponential decays, indicates that the probed hIXs undergo mostly radiative recombination even at high excitation powers (Supplementary Note 7). Thus, in the absence of photo-assisted tunnelling, power-independent radiative decays are dominant for all electric fields.

The absolute quantum yield of hIXs will also be critically dependent on *E*_*z*_. With respect to purely interlayer species in heterobilayers, lower absolute yields are expected from hIXs at low *E*_*z*_ and low *P*_in_ due to their momentum-indirect nature. However, purely IXs are characterized by emission yields that are both power- and field-dependent, with a strong decay in power due to second-order effects. Thus, we expect that at high *P*_*in*_ and high *E*_*z*_, the absolute yields of hIXs and IXs would become comparable. Future work could be dedicated specifically to quantitative comparisons between the absolute quantum yields of far-propagating out-of-plane excitonic species among different platforms.

These features indicate that the hybrid species in WSe_2_ homobilayers are promising for further studies of the propagation of highly interacting excitons, as platforms with constant emission quantum yields are required for the realization of efficient devices based on excitonic transport.

### Time-resolved transport properties of tunable hIXs

**Fig. 4:**
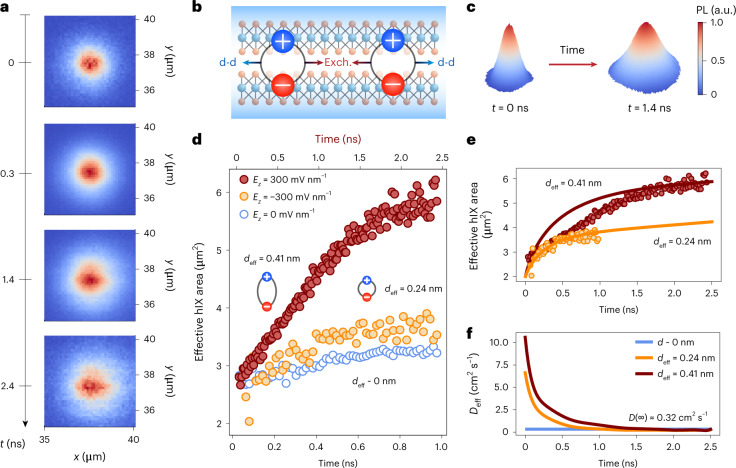
Time-resolved transport properties of tunable hIXs. **a**, Spatial imaging of high-dipole hIX cloud expansion at different *t* with respect to the laser pulse arrival obtained at *E*_*z*_ = 300 mV nm^−1^. **b**, Schematic representation of repulsive dipolar interactions (d-d) and attractive exchange forces (Exch.) between out-of-plane ensembles in a van der Waals homobilayer. **c**, 3D representation of the evolution of the measured hIX transporting cloud from *t* = 0 ns to *t* = 1.4 ns. **d**, Extracted effective exciton diffusion area based on the 1/*e* threshold as a function of time measured at different electric fields. The extracted laser area with respect to time is plotted in Supplementary Note 8 as a reference. Anomalous diffusion is observed for hIXs at high positive and negative electric fields. A linear expansion is obtained for negligible electric fields, indicating classical transport for excitons with low interlayer mixing. **e**, Theoretically computed hybrid exciton diffusion area for different levels of interlayer mixing (equivalent to different effective dipole moment lengths) calculated using the exciton density *n*_hiX_ and *D* extracted from experimental results. **f**, *D*_eff_ extracted from transport simulations with estimated maximum values of $D_{0.24{{{\mathrm{nm}}}}}^{\mathrm{MAX}} \approx 7\,{{{\mathrm{cm}}}}^2\,{{{\mathrm{s}}}}^{ - 1}$ and $D_{0.41{{{\mathrm{nm}}}}}^{\mathrm{MAX}} \approx 11\,{{{\mathrm{cm}}}}^2\,{{{\mathrm{s}}}}^{ - 1}$.

To fully understand the nature of interactions between propagating electrically tunable dipolar ensembles (Fig. 4b), we studied time-dependent hybrid exciton transport in our structure. To this purpose, we excited our sample with a picosecond pulsed laser and imaged the spatiotemporal expansion of the exciton cloud by its PL emission using a scanning avalanche photodiode system (Supplementary Note 8 and Methods)^14,41^. The spatially resolved exciton cloud corresponding to the high-*d* species is shown in Fig. 4a for different points in time. The effective hIX area as a function of time is reported for high-*d* and low-*d* ensembles in Fig. 4d. The equation of motion for the spatially resolved IX density is derived as (Supplementary Note 9):

2 $$\partial _tn\left( {{{{\bf{r}}}},t} \right) = D\nabla ^2n\left( {{{{\bf{r}}}},t} \right) + \mu _m\nabla \cdot \left( {\nabla \left( {{{\Delta }}E\left( {n\left( {{{{\bf{r}}}},t} \right)} \right)} \right)n\left( {{{{\bf{r}}}},t} \right)} \right) - \frac{{n\left( {{{{\bf{r}}}},t} \right)}}{\tau }$$

which takes the form of a 2D drift–diffusion equation, where *D* is the diffusion coefficient, *μ*_m_ = *D*/*k*_B_*T* is the exciton mobility, *k*_B_ is the Boltzmann constant, *T* is temperature and *τ* is the exciton lifetime. The energy renormalization term from equation (1) is now variable in space (**r**) and time (*t*) through *n*(**r**, *t*). Dipolar repulsions in the hIX equation of motion (equation (2)) cause a nonlinear response to a pump excitation in the form of anomalous diffusion (Fig. 4c). We therefore introduced an effective diffusivity term *D*_eff_(*t*), defined as the rate of change of the hIX cloud area (Supplementary Note 9). In hIX transport dynamics, we can distinguish between two main regimes at short (*t* < 1 ns) and long (*t* ≫ 1 ns) times after the laser pulse. The former regime is density-dependent anomalous diffusion, taking place when the hIX density is large. Here, *D*_eff_(*t*) is significantly enhanced with respect to the intrinsic *D* due to the interacting nature of hIXs (Supplementary Note 9). At longer time delays from the laser pulse, as the hIX density decreases, transport is dominated by conventional diffusion and *D*_eff_(*t*) converges to the intrinsic *D*.

Figure 4e,f shows the time evolution of the simulated hIX area and effective diffusivity for the dipole lengths of interest. The initial exciton density in transport simulations was estimated on the basis of a best-fit approach to the experimental results as $n_0 \simeq 10^{12}\,{{{\mathrm{cm}}}}^{ - {{{\mathrm{2}}}}}$ (Supplementary Note 9), below the exciton Mott transition limit in our system^42^. As the highest exciton densities in the spot area were obtained for *n*(**r**, 0), the maximum hIX effective diffusivity was found at the limit of *t*→0 for all dipolar species. Increasing $D_{{{{\mathrm{eff}}}}}^{\mathrm{MAX}}$ values were obtained for ensembles with higher *d*_eff_, with $D_{0.24{{{\mathrm{nm}}}}}^{\mathrm{MAX}} \approx 7\,{{{\mathrm{cm}}}}^2\,{{{\mathrm{s}}}}^{ - 1}$ and $D_{0.41{{{\mathrm{nm}}}}}^{\mathrm{MAX}} \approx 11\,{{{\mathrm{cm}}}}^2\,{{{\mathrm{s}}}}^{ - 1}$ estimated from our simulations (Fig. 4e,f). We note that the simulated transport for high-*d* ensembles produced a faster initial anomalous diffusion regime than the experimental data (*t* < 1 ns), thus causing an overshoot of the resulting effective diffusivity. Instead, good agreement between theoretical and measured data was reached in the low-*d* case. Therefore, given the trends observed in the anomalous diffusion regime, we concluded that both high-*d* and low-*d* species show similar maximum effective diffusivities in the range of 5–10 cm^2^ s^−1^. These diffusivity values correspond to an upper range of effective exciton mobility reaching $\mu _{{{{\mathrm{eff}}}}}^{\mathrm{MAX}} \approx 10,000\,{{{\mathrm{cm}}}}^2\,{{{\mathrm{V}}}}^{ - 1}\,{{{\mathrm{s}}}}^{ - 1}$ for high hIX densities and high-*d* transitions in the regime of anomalous diffusion.

The effective diffusivities of all probed excitons decreased monotonically in time, progressively saturating to $D_{{{{\mathrm{eff}}}}}\left( \infty \right) = D$, towards a regime of conventional diffusion. We note that $D_{{{{\mathrm{eff}}}}}\left( \infty \right)$ is independent of the effective dipole length as it is equivalent to the conventional diffusivity for an exciton gas at low excitation densities. The unaltered *D* was experimentally estimated by extracting the effective diffusivity of the hIX with a minimal interlayer character. Thus, by measuring the propagation of exciton ensembles at *E*_*z*_ = 0 mV nm^−1^, we obtained a conventional diffusivity of $D \simeq 0.32\,{{{\mathrm{cm}}}}^2\,{{{\mathrm{s}}}}^{ - 1}$ (Supplementary Note 10).

## Conclusions

Out-of-plane dipolar ensembles of optical excitations travel long distances owing to the strength of their repulsive forces, governed by the effective interlayer dipole length. In this work we achieved control over the layer hybridization of exciton states in a van der Waals homobilayer structure, allowing us to tune the effective dipole length of exciton ensembles. We characterized the dipole-dependent propagation of hIXs by modulating the interplay between attractive exchange interactions and repulsive Coulomb forces, the many-body effects governing exciton transport. The recorded lifetimes of hIXs in WSe_2_ homobilayers were lower than those reported for purely interlayer species (~1–600 ns)^6,11,13^. However, hIXs are characterized by an intrinsic diffusivity of $D_{\mathrm{hIX}} \simeq 0.3\,{{{\mathrm{cm}}}}^2\,{{{\mathrm{s}}}}^{ - 1}$, which is twice the diffusivity estimated for long-dipole excitons in moiré-less MoSe_2_/hBN/WSe_2_ heterotrilayers^14^, and significantly higher than those measured for MoSe_2_/WSe_2_ heterobilayers at 4 K (ref. ^23^). We also characterized the field-tunable regime of anomalous diffusion by spatiotemporally resolved measurements. Our study revealed a peak effective diffusivity of ~10 cm^2^ s^−1^, corresponding to an effective exciton mobility in the range $\mu _{{{{\mathrm{eff}}}}}^{\mathrm{MAX}} \approx 10{,}000\,{{{\mathrm{cm}}}}^2\,{{{\mathrm{V}}}}^{ - 1}\,{{{\mathrm{s}}}}^{ - 1}$.

The main factors affecting the efficiency of future interconnects and circuits based on exciton transport in van der Waals heterostructures are determined by the material absorption, the exciton mobility and the emission quantum yield. We have obtained power-independent high emission quantum yields of long-range propagating dipolar ensembles, a crucial step towards the efficient modulation of light in excitonic devices based on 2D materials. Furthermore, we exploited the spatiotemporal expansion of hybrid species to demonstrate that tunable repulsive dipolar exciton–exciton interactions can be achieved. This tunability makes WSe_2_ homobilayers highly attractive for future studies of strongly interacting bosonic systems.

Our microscopic understanding and control of the many-body effects governing the transport of dipolar exciton ensembles open avenues toward exploring exciton condensates in van der Waals structures and the realization of efficient excitonic devices based on 2D materials.

## Methods

### Device fabrication

All the devices used in this work comprised bottom gates fabricated using electron-beam lithography and metal evaporation (2 nm Cr/5 nm Pt) over a SiO_2_/Si substrate with an oxide thickness of 270 nm. The heterostructures in devices B and C were fabricated with a polymer-assisted wet transfer method. WSe_2_ (HQ Graphene) and hBN flakes were exfoliated on a polymer double layer, and bilayer WSe_2_ flakes were identified by atomic force microscopy measurements. The bottom polymer layer of the substrate was dissolved using a solvent, and the top polymer layer, together with the exfoliated flakes, was left free-floating. The bottom hBN layers, WSe_2_ bilayer and the top hBN layers were then carefully aligned and transferred in sequence on top of the bottom gates by using a dedicated home-built transfer set-up with motorized micromanipulators. The heterostructure of device A was fabricated using a dry-transfer technique employing polycarbonate membranes. WSe_2_ (HQ Graphene) and hBN flakes were exfoliated on a SiO_2_ substrate, identified by optical contrast and subsequently picked up using a single polycarbonate membrane. The heterostack was then released onto the Cr/Pt bottom gate by progressive adhesion following a temperature gradient above 150 °C. This dry-transfer technique allowed us to obtain large-area structures by avoiding contamination with polymer residues and water droplets. The optical images and atomic force microscopy measurements of the devices are shown in Supplementary Note 1. All of the heterostructures were annealed under a high vacuum (10^−6^ mbar) for 6 h at a temperature of 340 °C. Top gates and electrical contacts were then fabricated by electron-beam lithography and evaporation of Pt (4 nm) and Ti/Au (2 nm/80 nm) layers, respectively.

### Optical measurements

All optical measurements were performed in a vacuum at 4.6 K, unless stated otherwise, in a He-flow cryostat. Hybrid excitons were excited with a confocal microscope, while the emitted photons were collected through the same objective that had a working distance of 4.5 mm and a numerical aperture of 0.65. Optical pumping was achieved with a continuous-wave 640 nm diode laser (PicoQuant, LDH-IB-640-M) focused to the diffraction limit (spot full-width at half-maximum of 1.2 μm) for steady-state measurements. For micro-photoluminescence (μPL) spectral measurements, the emitted light was filtered by a 650 nm long-pass edge filter and then acquired using a spectrometer (Princeton Instruments SpectraPro 500) and recorded with a CCD (charge-coupled device) camera (Princeton Instruments, Blaze 400-HR/HRX). Spatial imaging of the IX emission was performed using a CCD camera (Andor Ixon) with an 800 nm long-pass edge filter that removed both the laser line and the intralayer emission from WSe_2_. For time-resolved measurements, the same solid-state laser was driven in a pulsed mode, achieving pulse widths lower than 160 ps at a repetition rate of 80 MHz. The collected photons were sent to an avalanche photodiode (APD, Excelitas Technologies, SPCM-AQRH-16) mounted on a 2D motorized translational stage. The output of the APD was connected to a time-correlated photon-counting module with a resolution of 12 ps r.m.s. (PicoQuant, PicoHarp 300), which measured the arrival time of each photon. We set the time bin to 16 ps for the measurements presented in this work. The single-photon timing resolution of the APD is ~350 ps, which is the main time limitation for this set-up. The technical details can be found in Supplementary Note 8.

### Microscopic many-particle theory

To study hybridized exciton states and anomalous exciton transport at elevated electron–hole densities in TMDC bilayers, we derived a many-body Hamiltonian on a hybrid exciton basis that contained a kinetic part and a part due to exciton–exciton interactions. By solving the bilayer Wannier equation, we gained access to pure intra- and interlayer exciton states. These were used as input to a hybrid exciton eigenvalue problem that accounted for mixing between intra- and interlayer exciton states. We found that momentum-dark KΛ (K′Λ′) excitons represented the energetically lowest-lying exciton states in naturally stacked WSe_2_ bilayers. We included an out-of-plane electric field by exploiting the Stark shift of the IX resonance, allowing us to tune the exciton landscape in bilayers as a function of the electrical field (Supplementary Note 2). The obtained hybrid exciton states were then used to compute density-dependent energy renormalizations obtained via the Heisenberg equation of motion (Supplementary Note 5). By allowing the exciton density to be spatially dependent, we found that the exciton–exciton interaction acts as a source to a drift term in a drift–diffusion equation. We gained access to the spatiotemporal dynamics of hybrid excitons by solving the drift–diffusion equation for hybrid excitons with different levels of field-driven interlayer mixing corresponding to different effective dipole moment lengths (Supplementary Note 9).

## Online content

Any methods, additional references, Nature Portfolio reporting summaries, source data, extended data, supplementary information, acknowledgements, peer review information; details of author contributions and competing interests; and statements of data and code availability are available at 10.1038/s41566-023-01198-w.

## Supplementary information

Supplementary Information Supplementary Notes 1–10, Figs. 1–11 and references.

## Data Availability

The data that support the findings of this study are available via Zenodo at 10.5281/zenodo.7660668.
