# Supplementary material for: Electrical control of hybrid exciton transport in a van der Waals heterostructure
Source: Nat Photonics. 2023 Apr 20;17(7):615–21. doi: 10.1038/s41566-023-01198-w (PMC10322698; doi:10.1038/s41566-023-01198-w)
Supplement: Supplementary file 1 — Supplementary Notes 1–10, Figs. 1–11 and references. [file 41566_2023_1198_MOESM1_ESM.pdf]

nature photonics

Article https://doi.org/10.1038/s41566-023-01198-w

Electrical control of hybrid exciton transport in a van der Waals heterostructure

In the format provided by the authors and unedited

1. Structure, morphology and uniformity of homobilayer WSe_2 devices..........................2
2. Exciton landscape in 2H-stacked WSe_2 bilayers..6
3. Dipole length extraction...9
4. Doping and asymmetrical Stark shifts in WSe_2 homobilayer devices.........................11
5. Hybrid exciton-exciton interaction and energy renormalizations................................13
6. Measured energy shift saturation...16
7. Lifetime measurements and constant emission quantum yield of hIXs 18
8. Experimental setup and optical measurements...22
9. Drift-diffusion equation and transport simulations..24
10. Estimation of the conventional diffusivity of hIXs..26

References...27

1. Structure, morphology and uniformity of homobilayer WSe_2 devices

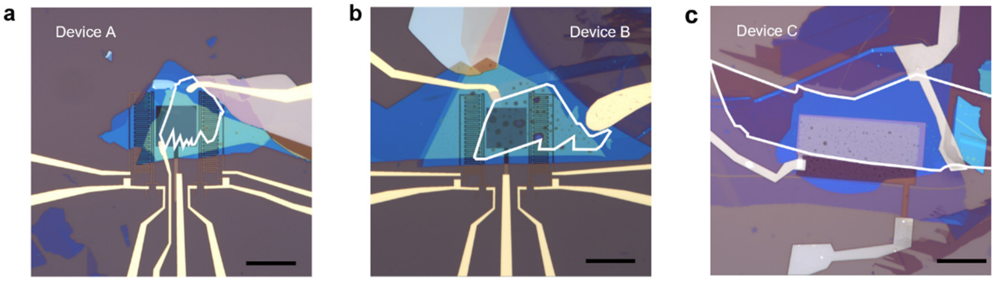

Figure S1. a-c, Optical images of the fully hBN-encapsulated WSe_2 homobilayer devices A, B, C. The WSe_2 homobilayers are highlighted by white contour lines. The metal contacts to the WSe_2 layers and to the top and bottom gates are clearly visible. Scale bar, 25 μm.

The fully hBN-encapsulated samples of devices A, B and C have been fabricated as described in the Methods section of the main text. In **Figure S1**, we show the optical images of the three devices with false-color white overlays indicating WSe_2 layers within the heterostacks. The bottom and top hBN layer thicknesses in devices A, B, C have been measured by AFM scans as 34 nm and 37 nm, 31 nm and 31 nm, 8.1 nm and 14.7 nm, respectively.

The AC-mode AFM topography maps of the active areas of devices A and B before top gate deposition are reported in **Figure S2**. A significant improvement in surface uniformity is obtained in device A by employing a dry-transfer technique, where instead device B was fabricated through a wet-transfer method, known to be more prone to the presence of bubbles and contaminations between layers of van der Waals heterostacks[1]. Thus, a flat clean area greater than 80 μm^2 is seen in device A from **Figure S2**. In particular, **Figure S2**c shows a detail of approximately 20 μm^2 where only small blisters are observed, where we define small blisters as bubbles of approximately 1 nm in height or lower.

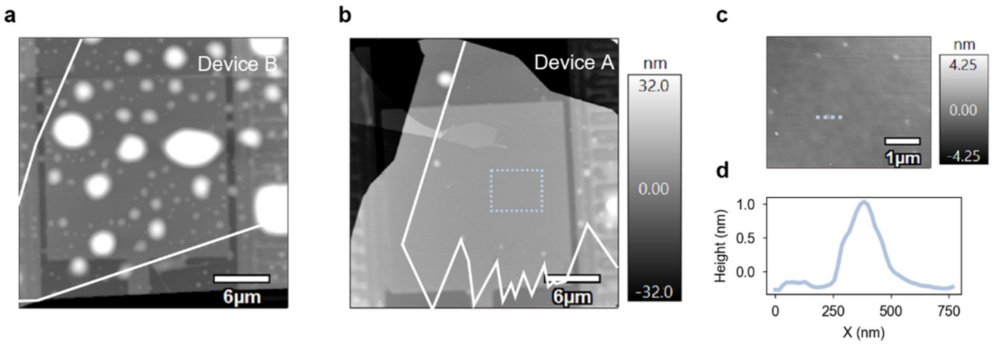

Figure S2. AC-mode AFM scans of the gated active areas of devices A (**b**) and B (**a**). The bottom gate is seen as a rectangular feature in the center of both scans. The same height ranges are used between the two AFM measurements in order to show the difference in morphology between device A, obtained by a dry-transfer technique, and device B, fabricated with a wet-transfer method. **c,** Close-up AFM scan of a portion of the central gated active area of device A, highlighted as a dashed light blue rectangle in (b). Only small blisters of approximately 1 nm in height are observed along the reported $4\mu m \times 5\mu m$ scan. **d,** Height measurement of a small blister, as highlighted in (c) with a shaded light blue line.

In Figure S3, we show the complete PL spectra for three different electric fields, explicitly indicating the presence of the purely-intralayer exciton species (X) at E≃1.72 eV. The transitions and phonon replicas of the hybrid species (1.48 eV<E<1.61 eV) have also been highlighted.

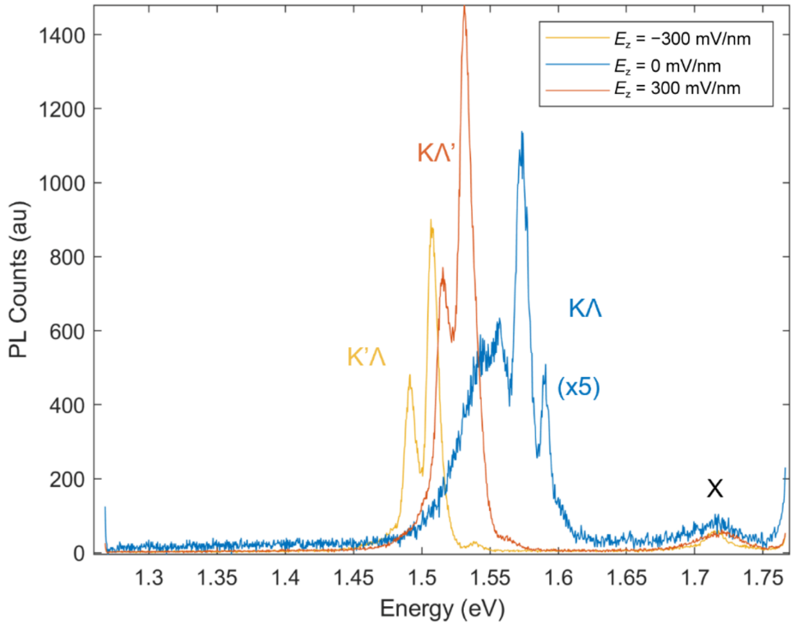

Figure S3. Low-temperature PL in 2H-stacked WSe_2 bilayers with the dominant transitions indicated for each electric field.

Obtaining large areas with uniform excitonic properties is crucial for reliable studies on propagating dipolar gases, as well as for envisioning the realization of large-scale excitonic circuits based on van der Waals heterostructures. In this section, we show that the clean spatial conformation of device A is directly related to a low dispersion of the excitonic properties within the active area of interest.

For this purpose, we have first performed PL maps throughout the clean device area shown in Figure S4b. The map steps are of 1 µm conforming to the dimensions of the laser beam used in this work (see Methods). All measurements are performed at a fixed field of $F_{el} = 300\ \mathrm{mV/nm}$, where the PL emission of the $K\Lambda'$ transition is mainly characterized by two peaks due to phononic replicas. We will refer to P1 and P2 as the peaks with highest and lowest energy in the spectrum, respectively (Figure S4c). Within the 80 μm^2 dual-gated active area of interest, P1 and P2 are centered at average energies of $E^{P1} = 1.5237$ eV and $E^{P2} = 1.5068$ eV, with standard deviations of $\sigma_E^{P1} \simeq \sigma_E^{P1} = 0.9$ meV. The average linewidth full-width half maximum values are found to be $FWHM^{P1} = 14.4$ meV and $FWHM^{P2} = 10.2$ meV, with deviations of $\sigma_{FWHM}^{P1} = 1.6$ meV and $\sigma_{FWHM}^{P2} = 0.6$ meV. The dispersion of the peak energy position of the transition of interest is more than one order of magnitude lower than its linewidth, meaning that an ideal uniformity of PL emission is obtained throughout the area of interest. Thus, we can consider the excitonic characterization in our device to be independent on the position within the structure.

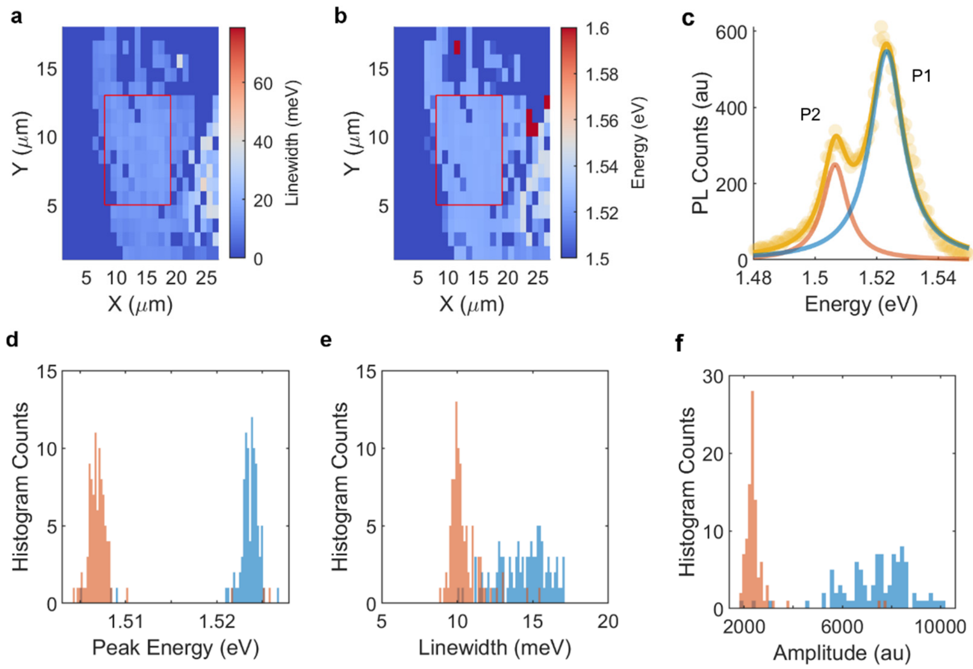

Figure S4. a-b, Spatial maps of the full-width half-maximum (FWHM) (a) and peak energy position (b) in device A for the highest-energy PL peak at $E_z = -300\,\mathrm{mV/nm}$. **c,** PL emission (yellow) fitted for two main peaks, P1 (blue) and P2 (red). **d-f,** Statistics of the peak energy (d), FWHM I and amplitude (f) of P1 (blue) and P2 (red) throughout the $80\mu\mathrm{m}^2$ area highlighted in red in (a) and (b). The average peak energies are $E^{P1} = 1.5237\mathrm{eV}$ and $E^{P2} = 1.5068\mathrm{eV}$, with standard deviations of $\sigma_E^{P1} \simeq \sigma_E^{P2} = 0.9\mathrm{meV}$. The linewidths have averages of $FWHM^{P1} = 14.4\mathrm{meV}$ and $FWHM^{P2} = 10.2\mathrm{meV}$, with deviations of $\sigma_{FWHM}^{P1} = 1.6\mathrm{meV}$ and $\sigma_{FWHM}^{P2} = 0.6\mathrm{meV}$. The average amplitudes in arbitrary units are $I^{P1} = 7553$ and $I^{P2} = 2357$, with $\sigma_I^{P1} = 1156$ and $\sigma_I^{P2} = 283$. Higher dispersions for the highest-energy peak FWHM and amplitude are observed. Instead, similar statistics are obtained for the energy peak position of P1 and P2.

2. Exciton landscape in 2H-stacked WSe_2 bilayers

We obtain the exciton landscape of transition-metal dichalcogenide bilayers by first solving the bilayer Wannier equation[2]. This gives us microscopic access to pure intra- and interlayer exciton states. Furthermore, we take into account the effect of hybridization of intra- and interlayer exciton states via electron/hole tunneling by solving the hybrid exciton eigenvalue equation, as discussed in detail in Ref. [3]. The relevant input parameters including the electron/hole tunneling strengths can also be found in there. In our theoretical framework, we explicitly take into account electrons in the vicinity of the $\xi^e = K, K', \Lambda, \Lambda'$ valleys and holes in the vicinity of the $\xi^h = K, K'$ valleys within the effective mass approximation when we solve the eigenvalue equation for hybrid excitons and focus on spin-bright transitions. In Table S1, we summarize the obtained (hybrid) exciton energies and corresponding intralayer and interlayer mixing coefficients given by $|C_X|^2$ and $|C_{IX}|^2$ for hBN-encapsulated 2H-stacked WSe_2 bilayers in the absence and presence of an electric field, $|E_z| = 300$ mV/nm.

Exciton (ξ^h, ξ^e)	Energy (meV)	$\lvert C_X\rvert^2$	$\lvert C_{IX}\rvert^2$
$K\Lambda$	-123/**-152.3**/-209	0.85/**0.77**/0.61	0.15/**0.23**/0.39
$K'\Lambda'$	-209/**-152.3**/-123	0.61/**0.77**/0.85	0.39/**0.23**/0.15
$K\Lambda'$	-11.5/-109.6/**-250**	0.58/0.36/**0.2**	0.42/0.64/**0.8**
$K'\Lambda$	**-250**/-109.6/-11.5	**0.2**/0.36/0.58	**0.8**/0.64/0.42

Table S1. Exciton landscape in hBN-encapsulated 2H-stacked WSe_2 homobilayers, obtained by microscopically solving the eigenvalue equation for hybrid excitons. The three numbers in columns 2-4 refer to the cases $E_z = -300,0,300$ mV/nm with the energetically lowest transitions marked in bold and given relative to the KK intralayer exciton energy. For vanishing electric fields we find that the $K\Lambda$ state is the energetically lowest state.

The mixing coefficients are obtained from solving the hybrid exciton eigenvalue equation[4] and crucially determine the intra/interlayer mixing of the hIX. Due to the reversed spin-orbit coupling in one of the TMDC layers, the energetically lowest exciton states are degenerate and represented by excitons stemming from opposite valleys (i.e. by e.g. $\xi = KK$ and $\overline{\xi} = K'K'$) in H-type TMDC homobilayers. Furthermore, these state have opposite dipole moments[6]. The relative energetic position of intra- and interlayer exciton states can be tuned by applying an electric field in the out-of-plane direction. In particular, the energy of interlayer exciton states is shifted by the electric field as these excitons exhibit a permanent out-of-plane dipole moment. In our model, this is included by introducing a Stark shift $e_0 d E_z$ in the hybrid exciton eigenvalue equation[6], where e_0 is the electronic charge, d is the interlayer charge separation

(approximately equal to the TMDC layer thickness, here taken as 0.65 nm). In Figure S5 we illustrate the exciton landscape in 2H-stacked WSe_2 homobilayers as function of an out-of-plane electric field. Importantly, we note that the interlayer mixing coefficient is increased (decreased) when applying an electric field parallel (anti-parallel) to the dipole moment of the hIX (Figure S5b). At vanishing electric fields, we find that the phonon-assisted emission in 2H-stacked WSe_2 stem primarily from $K\Lambda$ excitons with small interlayer components (approximately 20%), as also evidenced by recent DFT calculations[7,8]. In contrast, at large positive electric fields $K\Lambda'$ excitons dominate the phonon-assisted emission. These states possess a large interlayer mixing coefficient (approximately 80 %), which couple more strongly to electric fields.

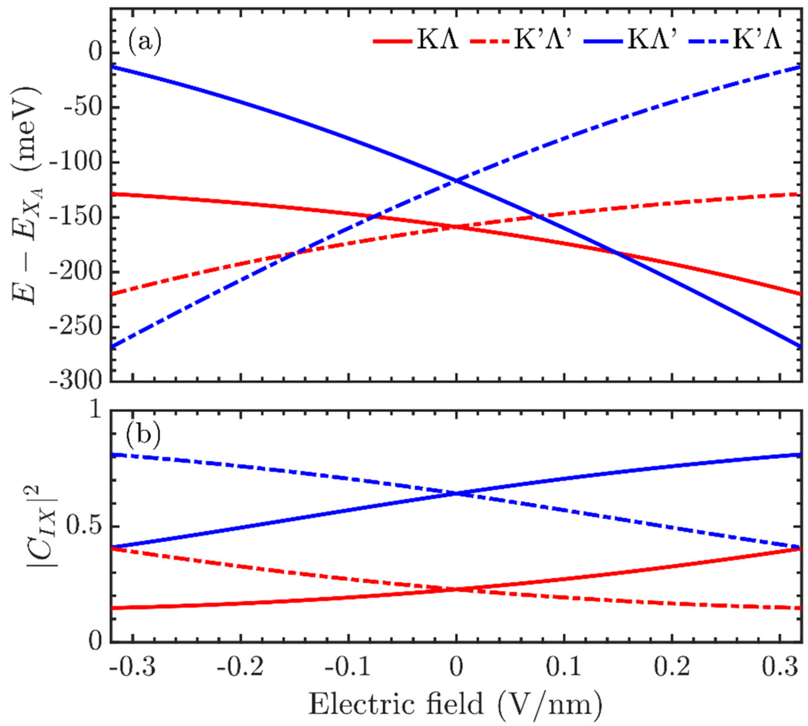

Figure S5. Exciton landscape in 2H-stacked hBN-encapsulated WSe_2 homobilayers as a function of electrical field, E_z. **a,** Hybrid exciton energies given relative to the intralayer A exciton energy (X_A). **b,** Interlayer mixing coefficients for $K\Lambda$ ($K'\Lambda$) and $K\Lambda'$ ($K'\Lambda$) excitons.

Dark (hybrid) excitons are visible in photoluminescence spectra via phonon sidebands at low temperatures (Figure 1d in the main text). The dominating transitions have been identified in the discussion above and labelled in Figure S5. Interestingly, the PL spectrum displays changes

of the spectral weights, linewidths, and number of peaks as a function of the external electric field. The spectra in the case of negative electric field reflects well the theoretically predicted PL spectra in a similar TMDC structure based on the same material[5], where the two peaks arise from recombination channels involving optical and acoustic phonons. In particular, the peak separation (~ 20 meV) is in good agreement with the energy difference between optical and acoustic phonons at the Λ-point for WSe_2 monolayers[9]. On the other hand, the cases of vanishing and positive electric fields display a larger number of peaks with various linewidths and spectral weights. Such features could result from the presence of intrinsic doping in these cases, which is known to give rise to additional (trion) peaks, modified spectral weights and enhanced linewidths in the PL spectra[10]. Although a microscopic understanding of these features is desirable, it is out of the scope of this work.

3. Dipole length extraction

In order to estimate the vertical electric field within the active homobilayer area as a function of the applied top and bottom gate voltages, we use an effective value for the permittivity of the active heterostructure, which can be considered to be equal to that of bilayer WSe_2. Thus, the heterostructure permittivity ε_{hs} is taken as[11]:

$$\varepsilon_{hs} = \varepsilon_{2L-WSe_2} = 7.5 \tag{1}$$

We refer to the vertical electric field as E_z, which can be computed as a function of the top and bottom gate voltages, V_{tg} and V_{bg}:

$$E_z = \frac{V_{bg} - V_{tg}}{d_{tot}} \cdot \frac{\varepsilon_{hBN}}{\varepsilon_{hs}} = \frac{V_{DIFF}}{d_{tot}} \cdot \frac{\varepsilon_{hBN}}{\varepsilon_{hs}} \tag{2}$$

where $\varepsilon_{hBN} \approx 4$ and $d_{tot} = d_{hBN}^{top} + d_{hBN}^{bot} + d_{WSe_2}$. In the case of device A, we use $d_{hBN}^{top} =$ 37 nm, $d_{hBN}^{bot} = 34$ nm, $d_{WSe_2} = 1.3$ nm. The energy shift for excitons with a sizeable out-of-plane dipole d and an applied vertical electric field E_z is given by the quantum-confined Stark effect:

$$\Delta E_{hIX}(E_z) = E_{hIX}(E_z) - E_0 = |edE_z| \tag{3}$$

By taking the derivative of both sides with respect to E_z we obtain:

$$\left|\frac{\partial \Delta E_{hIX}(E_z)}{\partial E_z}\right| = \left|\frac{\partial \Delta E_{hIX}(E_z)}{\partial E_z}\right| = |ed| \tag{4}$$

Finally, the dipole length d is estimated as:

$$d = \left|\frac{1}{e} \cdot \frac{\partial \Delta E_{hIX}(E_z)}{\partial E_z}\right| \tag{5}$$

where $\partial \Delta E_{hIX}(E_z)/\partial E_z$ is the variation of the peak emission energy shift with respect to the applied electric field.

In order to determine the dipole moment as in Eq. (5), we measure the spectral emission of exciton species at a fixed excitation power, while varying the top and bottom gate voltages. We can induce a vertical electric field with no electrostatic doping from gating, applying $V_{tg} = -\alpha V_{bg}$, where $\alpha = h_{tBN}/h_{bBN}$ is the ratio between the thicknesses of the top and bottom hBN layers in the device structure (Supplementary Note 1). To avoid the overlap of different excitonic features when extracting effective dipole lengths d_{eff}, we define distinct regimes of predominant transitions belonging to either $K\Lambda$ ($K'\Lambda'$) or $K\Lambda'$ ($K'\Lambda$) where a single excitonic specie is dominant in the PL spectra. Then, we linearly fit the change in peak emission energy with respect to the applied vertical field within each range in order to estimate the value of d_{eff} for hIXs with different levels of hybridization. The ranges of E_z used for the dipole length estimation in Figure 1f are [-0.4; -0.2] V/nm, [-0.15; -0.05] V/nm, [0.15; 0.2] V/nm and [0.3; 0.4] V/nm, yielding estimated d_{eff} values of 0.24 nm, 0.13 nm, 0.11 nm, 0.41 nm, respectively.

4. Doping and asymmetrical Stark shifts in WSe_2 homobilayer devices

Device C was fabricated with the aim of studying the relationship between hIX dipole length and electrostatic doping. In field-effect structures, thin dielectric layers are preferred for the purpose of enhancing the electrostatic control over charge accumulation in the semiconducting layers. Therefore, the heterostructure of device C features 8.1 nm and 14.7 nm thick bottom and top hBN, respectively, allowing broader control over the gate-induced electrostatic doping within a reduced range of gate voltages with respect to devices A and B. In order to apply a vertical electric field and electrostatically dope the WSe_2 layers at the same time, we fix the relationship between the applied top and bottom gate voltages as:

$$V'_{tg} = -V_{tg} + \alpha V_{OFF}$$
$$V'_{bg} = V_{bg} + V_{OFF} \tag{6}$$

where $\alpha = h_{tBN}/h_{bBN}$, and V_{OFF} is an offset voltage to induce a fixed electrostatic doping level to the WSe_2 bilayer. The estimation of the vertical electric field in the homobilayer E_z is conducted using the values of V_{tg} and V_{bg} as shown in Supplementary Note 3, with $V_{tg} = -\alpha V_{bg}$. Comparing electric field sweeps at different doping voltages, a change in the slope of the Stark shift of the main excitonic emission is evident (Figure S6c). The dipole moment is then extracted from E_z for different offset voltages, corresponding to different levels of electrostatic doping (Figure S6f).

From Figure S6, we establish that the dipole length of the probed collective dipolar ensembles belonging to high-d transitions can be strongly modulated by an applied offset voltage within a range between 0.26 nm and 0.18 nm for a variation of V_{OFF} from -3 V to 3 V. Thus, we establish that electrostatic doping levels cause a shift in the effective out-of-plane dipole lengths of the probed excitons[12,13]. These findings, together with the behavior of the asymmetric $K\Lambda'$ and $K'\Lambda$ transitions discussed in the main text (Figure 1 and Figure 2), suggest that induced or intrinsic doping densities give different effective levels of layer hybridization, thus causing a change in the balance between Coulomb and exchange interactions in hIX dynamics with respect to an ideal WSe_2 bilayer. A deeper understanding of the microscopic origin of this characteristic behavior requires further studies which are out of the scope of this work.

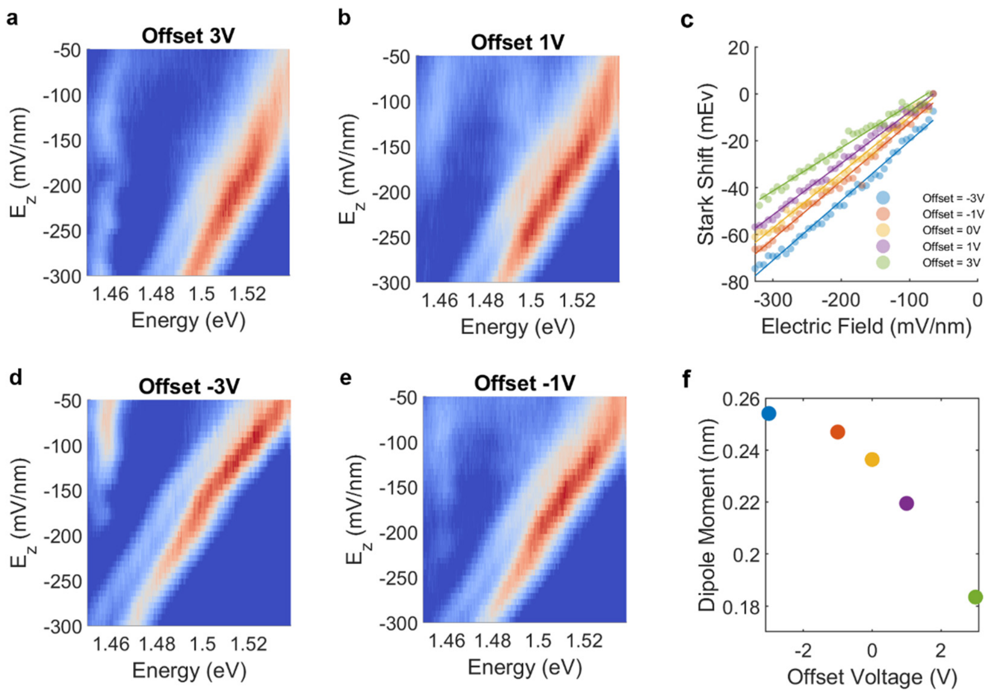

Figure S6. a,b,d,e Field-dependent PL spectra obtained from device C with fixed offset voltages V_{OFF} of 3V, 1V, -3V, -1V, respectively. We are focusing on a single branch of $K'\Lambda$ transitions ($F_{el} < -80\,\mathrm{mV/nm}$). **c,** Energy shift of the main PL peak for V_{OFF} ranging from -3V to 3V. A progressive change in the slope of the linear shifts is clearly visible. **f,** Dipole length as a function of V_{OFF}, extracted from (c) as described in Supplementary Note 3. A tunability of approximately 0.1 nm is obtained for the explored range of offset voltages.

5. Hybrid exciton-exciton interaction and energy renormalizations

In order to address density-dependent many-body renormalizations and non-linear hybrid exciton transport we derive the corresponding hybrid exciton-exciton Hamiltonian. The derivation of layer-hybridized intra- and interlayer exciton-exciton interaction can be found in Refs. [[3,14,15]]. Here, we generalize the approach in Ref. [[3]] to include multiple layers and valley species and obtain the Hamiltonian

$$H_{x-x} = \sum_{\substack{Q_1, Q_2, q, \\ \zeta_1, \zeta_2, \zeta_3, \zeta_4}} \widetilde{W}^{\zeta_1 \zeta_2 \zeta_3 \zeta_4}_{Q_1, Q_2, q} X^{\dagger}_{\zeta_1, Q_1+q} X^{\dagger}_{\zeta_2, Q_2-q} X_{\zeta_3, Q_2} X_{\zeta_4, Q_1} \tag{7}$$

where $\zeta_i = (\xi_i, L_i), \xi_i = \left(\xi_i^h, \xi_i^e\right), L_i = \left(l_i^h, l_i^e\right)$ is a compound index taking into account valley and layer degrees of freedom, respectively. The exciton-exciton interaction matrix can be expressed as $\widetilde{W}^{\zeta_1\zeta_2\zeta_3\zeta_4}_{Q_1,Q_2,q} = \widetilde{D}^{\zeta_1\zeta_2}_{q} \delta_{\zeta_1, \zeta_4} \delta_{\zeta_2, \zeta_3} + \widetilde{E}^{\zeta_1\zeta_2\zeta_3\zeta_4}_{Q_1,Q_2,q}$ with $\widetilde{D}$ and $\widetilde{E}$ being the direct and exchange part, respectively. Secondly, we hybridize the obtained Hamiltonian by transforming the exciton operators $X^{(\dagger)}$ to the hybrid basis such that $X_{\xi, L, Q} = \sum_\eta Y_{\eta, \xi, Q}\, C^{\eta}_{L, Q}$, where $Y^{(\dagger)}$is a new set of hybrid exciton operators, C is the mixing coefficient, and η enumerates the hybrid exciton state. By absorbing the mixing coefficients into a new matrix element, $W = \widetilde{W} * |\mathrm{C}|^4$, we obtain a hybrid exciton-exciton Hamiltonian. This Hamiltonian can be used together with the Heisenberg equation of motion of the excitonic polarization $\partial_t \left\langle Y^{(\dagger)}_{\xi,Q} \right\rangle = \frac{i}{\hbar}\left[H_{hyb,x-x}, Y^{(\dagger)}_{\xi,Q}\right]$ to compute the corresponding density-dependent spectral shift for a hybrid exciton on a Hartree-Fock level:

$$\Delta E^{\xi} = \left(W^{\xi\bar{\xi}\bar{\xi}\xi}_{0,0,0} + W^{\xi\bar{\xi}\xi\bar{\xi}}_{0,0,0}\right) n_x^{\bar{\xi}} + 2W^{\xi\xi\xi\xi}_{0,0,0} n_x^{\xi}, \tag{8}$$

where $n_x^{\xi} = \sum_Q \left\langle Y^{\dagger}_{\xi, Q} Y_{\xi, Q} \right\rangle$ is the valley-specific hybrid exciton density. Note that we focus here on the case of two competing valley species ξ and $\bar{\xi}$, with $\bar{\xi}$ being the opposite valley of ξ, i.e. if $\xi = KK$, then $\bar{\xi} = K'K'$. We note that the first two terms involve interactions between excitons in opposite valleys ($\xi \neq \bar{\xi}$) with opposite dipole moments. Moreover, the exchange part of the interaction is vanishing between exciton species in different valleys as electron-electron or hole-hole exchange cannot occur if the scattering charge constituents reside at different high symmetry-points[3,15,16]. We are then left with:

$$\Delta E^{\xi} = g_{d-d}^{\xi\bar{\xi}} n_x^{\bar{\xi}} + 2\left(g_{d-d}^{\xi\xi} + g_{x-x}^{\xi\xi}\right) n_x^{\xi}, \tag{9}$$

defining $W_{dir,\,0,0,0}^{\xi\xi\xi\xi} \equiv g_{d-d}^{\xi\xi}$, $W_{exch,\,0,0,0}^{\xi\xi\xi\xi} \equiv g_{x-x}^{\xi\xi}$, and $W_{0,0,0}^{\xi\bar{\xi}\bar{\xi}\xi} \equiv g_{d-d}^{\xi\bar{\xi}}$. While we use the equation above to compute the spectral shifts, we remark that the above formula can be further simplified in the presence of an electric field. First, considering the case without an electrical field it holds that $n_x^{\xi} = n_x^{\bar{\xi}}$ (as the states are energetically degenerate) simplifying the equation above to:

$$\Delta E_{E_z=0}^{\xi} = 2n_x^{\xi}\left(\frac{g_{d-d}^{\xi\xi}}{2} + g_{x-x}^{\xi\xi}\right), \tag{10}$$

using $g_{d-d}^{\xi\bar{\xi}} = -g_{d-d}^{\xi\xi}$ (as the excitons ξ and $\bar{\xi}$ exhibit opposite dipole moments, cf. Supplementary Note 2). Hence, the dipole-dipole contribution is weakened relative to the exchange contribution. In the presence of a large electrical field it holds that $n_x^{\xi} \gg n_x^{\bar{\xi}}$ such that:

$$\Delta E_{|E_z|\gg 0}^{\xi} = 2\left(g_{d-d}^{\xi\xi} + g_{x-x}^{\xi\xi}\right) n_x^{\xi}. \tag{11}$$

Finally, we provide the expressions for the direct (dipole-dipole, $g_{d-d}^{\xi\xi}$) and the exchange ($g_{x-x}^{\xi\xi}$) interaction strengths:

$$g_{d-d}^{\xi\xi} = \frac{de_0^2}{2\epsilon_0\epsilon^{\perp}}\left|C_{\xi,IX}\right|^4,\; g_{x-x}^{\xi\xi} = \sum_{k,k'}\left(V_{k-k'}^{IX}\varphi_{k'}^{\xi} - V_{k-k'}^{X}\varphi_{k}^{\xi}\right)\varphi_k^{*\xi}\left|\varphi_{k'}^{\xi}\right|^2\left|C_{\xi,IX}\right|^4 \tag{12}$$

Here, the interlayer mixing coefficients $C_{\xi,IX}$ crucially enter, together with the interlayer excitonic wave functions $\varphi_k$obtained from the Wannier equation. The mixing coefficients are highly sensitive to changes in the electric field (Table S1) and consequently also the exciton-exciton interaction strengths vary strongly with E_z . The intra- and interlayer Coulomb interactions V_k^X and V_k^{IX}, respectively, explicitly include the dielectric environment and the finite thickness of the TMDC layers via a generalized Keldysh screening[2,3]. Density-dependent screening effects such as exciton screening and free electron-hole screening have been neglected. Furthermore, we include the dominant interlayer exciton-exciton interactions and

neglect intralayer contributions. These contributions are known to result in minor energy redshifts with increased exciton density[3,17]. As both the dipole-dipole and the exchange contribution to the energy renormalization scale linearly with exciton density, we extract an effective dipole moment, d_{eff}, for the hIX according to $\Delta E = n_{hIX} \frac{d_{\mathrm{eff}}}{\epsilon_0\, \epsilon^{\perp}}$,where ϵ_0 is the vacuum permittivity and $\epsilon_{\perp}$ is the out-of-plane component of the dielectric tensor of the TMDC. This provides an easy way to quantify the full exciton-exciton interaction strength including both dipolar and exchange contributions.

6. Measured energy shift saturation

Density-dependent energy renormalization shifts have been calculated for an ideal WSe_2 structure with no doping (Figure 2d). By considering the effect of doping on the asymmetrical Stark shift tendencies, we model the energy renormalization with respect to n_{hIX} for effective dipoles of 0.41 nm and 0.24 nm (Figure 2c), conforming to the d_{eff} values extracted from the measured Stark shifts for device A (Figure 1f). Linear energy renormalization trends are obtained from calculations within a wide range of hIX densities, with different slopes based on the level of hybridization. On the other hand, the measured PL peak energy shifts are characterized by a linear increase for low excitation power ranges ($P_{in} < 150\mu\mathrm{W}$), followed by an abrupt saturation with a slight redshift for moderate to high powers. Previous reports on purely interlayer excitons in type-II heterostructures of TMDCs[18–20] have shown a sublinear behavior for both the measured blueshift and the full-width half-maximum of the main interlayer exciton peak with respect to power, together with a sublinear trend of the integrated PL intensity counts. These results comply with the fact that density-dependent recombination channels dominate the dynamics of IXs in type-II heterobilayers and monolayer hBN spaced heterobilayers, thus implying that the measured blueshift and PL intensity trends are both related to a sublinear increase of the IX density with power. Instead, we observe a linear increase in the integrated hybrid exciton PL intensity with respect to P_{in} (Figure 3b).

Even though we can exclude exciton-exciton annihilation as a reason for the energy shift saturation of hIX emission in our structure, we must consider other power-dependent phenomena to explain the observed behavior. Previous reports on indirect excitons in double quantum well structures have reported a linear blueshift at low excitation densities followed by an abrupt saturation[21]. By ruling out the common many-body effects involved in the exciton dynamics, lattice heating has been deemed responsible for the redshift at high indirect exciton densities in double quantum wells. In this study, our theoretical calculations predict density-dependent energy renormalization shifts by including dipolar and exchange interactions, which are both linearly dependent on n_{hIX}. Therefore, we exclude that the energy shift saturation is related to the interactions within the many-body system. Thus, we can fairly ascribe the shift saturation at high moderate-to-high exciton densities to a band-gap redshift at high laser power in the form of lattice heating.

Moreover, we note that the measured renormalization shift magnitudes are differ by one order of magnitude between the ensembles with 0.24 nm and 0.41 nm effective dipoles. The behavior of PL intensity with respect to excitation power (Figure 3b) indicates that n_{hIX} linearly increases with power. Moreover, given that the PL ratio between high-d and low-d species is ~ 2.5, and that both $n_{hIX} \propto G\ \tau$ and $I_{PL} \propto G\ \tau$, we must exclude a relationship between the renormalized energies and the values of n_{hd} and n_{ld} at high optical powers. Therefore, the range of exciton densities taken into account for the high-d and low-d ensembles is estimated based on transport simulations, as further detailed in Supplementary Note 9. We tentatively ascribe the difference in measured ΔE between the two dipolar ensembles to an effect of intrinsic doping, which is the main source of imbalance between the two high-field branches of the explored hybrid excitons (Supplementary Note 4).

7. Lifetime measurements and constant emission quantum yield of hIXs

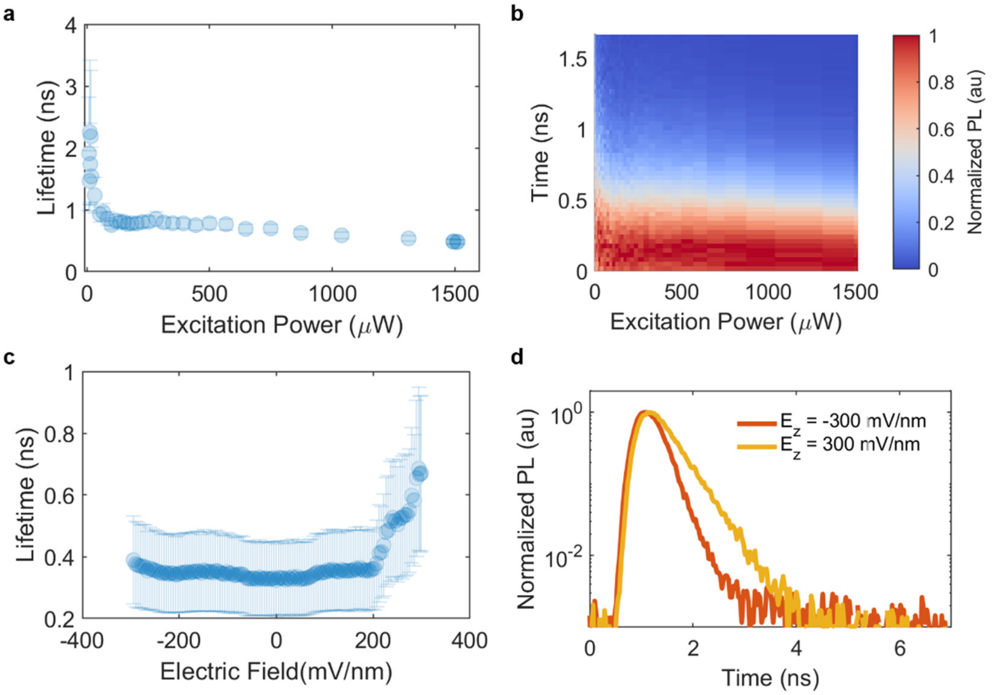

Figure S7. a, Power-dependent lifetime in device A at $E_z = -300\,mV/nm$. The lifetime is extracted from a single-exponential fit to time-resolved PL measurements. A monotonic decrease in τ_{tot} is observed for $20\mu W < P_{in} < 1500\mu W$. **b,** Power-dependent normalized PL intensity map with respect to time at $E_z = -300\,mV/nm$. **c,** Field-dependent hIX lifetime in device A, showing a similar behavior to that of device B (Figure 3a in the main text). **d,** Time-resolved PL signal for electric fields of $300\,mV/nm$ and $-300\,mV/nm$, corresponding to effective dipoles of 0.41 and 0.24 nm, respectively. Data in (a) and (c) are presented as mean values (dots) and corresponding standard deviations (error bars).

We have performed time-resolved measurements on WSe_2 homobilayer devices A and B. Both show a dependence with respect to the vertical electric field, with the minimum lifetime obtained for negligible electric fields and increasing values for higher E_z. The results from device B are reported in Figure 3a and Figure 3c in the main text. In Figure S7, we show time-resolved photoluminescence (TRPL) measurements at different input optical powers and applied electric fields for device A. Single-exponential decays are obtained independently of the field or power, suggesting that second-order recombination channels are intrinsically suppressed in this platform. Thus, the lifetime of the probed excitons is extracted as τ_{tot} by single-exponential fittings to the obtained TRPL data. High error dispersions are observed at very low power due to low signal-to-noise ratios (Figure S7a). The full data set is nonetheless

shown for consistency. Within the trustable range $P_{in} > 20\mu W$, we observe a monotonic decrease in lifetime ranging from approximately 0.8 ns to 0.4 ns at 1.5 mW.

The probed PL intensity I_{hIX} emitted from our devices is related to the hybrid exciton density n_{hIX} at a fixed electric field by the relationship $I_{hIX} = n_{hIX}\text{QY}$, where QY is the emission quantum yield of the probed species. We have shown in Figure 3b that the emitted PL intensity increases linearly with excitation power independently on the applied vertical electric field and on the hIX level of hybridization. Since TRPL measurements reveal that hIXs in WSe_2 bilayers are characterized by single-exponential decays, the exciton density can be related to the hIX generation factor G by $n_{hIX} = G\tau_{tot}$. The generation factor is related to the material absorption, as well as to the efficiency of hybrid exciton formation, and is directly dependent on the input optical power $G \propto P_{in}$. Since the change in τ_{tot} within the range of laser excitation power of interest is orders of magnitude lower than that of n_{hIX}, we can consider an approximately constant value $\tau_{tot}(P_{in}) \sim \tau_{tot}$. Thus, the linear increase in PL intensity at a fixed excitation power corresponds to a linear increase in n_{hIX} with respect to excitation power.

These observations indicate that a constant output QY is obtained for all probed hIX species independently on the level of hybridization. This behavior is significantly different than that of purely interlayer excitons in type-II heterobilayer and monolayer hBN spaced heterobilayers structures, where the emitted PL intensity has been shown to saturate at high excitation power due density-dependent nonradiative recombination channels[18,19,22,20], such as exciton-exciton annihilation[23]. Consistently, the previously reported TRPL signals from type-II IXs at high power can be better described by double-exponential decays due to fast decay channels from second-order density-dependent nonradiative recombination channels[22].

In order to determine the presence of power-independent nonradiative decay channels for purely-interlayer excitons in $WSe_2/MoSe_2$, Jauregui *et al.*[22] have investigated the relationship between lifetime and PL intensity with respect to the applied vertical electric field. In particular, it been shown for purely-interlayer excitons in $WSe_2/MoSe_2$ that a relationship between τ_{tot} and I_{hIX} with a negative slope is a direct indication of concurring τ_{rad} and τ_{nonrad} factors. We note that this way of proceeding is independent on the generation factor G, and thus can be applied to any platform with a field-tunable lifetime of excitons. The emission QY in our structure is given by the ratio between the τ_{tot} and the radiative lifetime τ_{rad}. Thus, we obtain:

$$I_{hIX} = \frac{\tau_{tot}}{\tau_{rad}} G\tau_{tot} \tag{13}$$

Both I_{hIX} and τ_{tot} are modulated by an applied vertical electric field (Figure 3a), with different trends based on the ranges of electric field intensity. Figure 3c shows that a linear relationship is present between I_{hIX} and τ_{tot} both for $K\Lambda$ and $K\Lambda'$ transitions. Since the excitation power is kept constant in field-dependent measurements, a linear relationship with positive coefficient is obtained in Eq. (13) only if $\tau_{tot} \simeq \tau_{rad}$. Thus, we conclude that the probed excitons are characterized by a constant QY with respect to power with mainly radiative recombination channels and suppressed nonradiative ones.

For all previous analyses, we have focused on the linear trends of τ_{tot} with respect to E_z belonging to regions of different predominant intervalley transitions in our sample. However, a sudden drop in τ_{tot} is observed in device B at high negative and positive electric field (Figure S8a), breaking the monotonicity of τ_{tot}. This behavior can be explained by considering the appearance of a nonradiative recombination channel at high electric fields in the form of tunneling photocurrent. Tunneling photocurrents have been studied in optically-excited field-effect devices based on semiconducting 2D materials[24–26], where different mechanisms based on photoexcitation can allow carriers to tunnel from the semiconductor to the gate electrode through dielectric barriers. The appearance of nonradiative channels at high electric fields have been previously observed in WSe_2 homobilayer devices[13]. Moreover, previous reports on spatially-indirect excitons in double quantum wells have revealed that the exponentially increasing dissociation rates of out-of-plane ensembles induce a sudden drop in lifetime at high electric fields[27,28]. From the measurements in device B, we observe an increasing tunneling photocurrent signal for high electric fields. In particular, Figure S8c shows the rising tunneling photocurrent for $E_z > 160\,\text{mV/nm}$, corresponding to the region of drop in τ_{tot} for positive fields (Figure S8b). Thus, it is reasonable to conclude that in our case hIXs dissociate at high electric fields, and the separated carriers tunnel through the hBN barriers to the gate electrodes inducing the measured tunneling photocurrent signal.

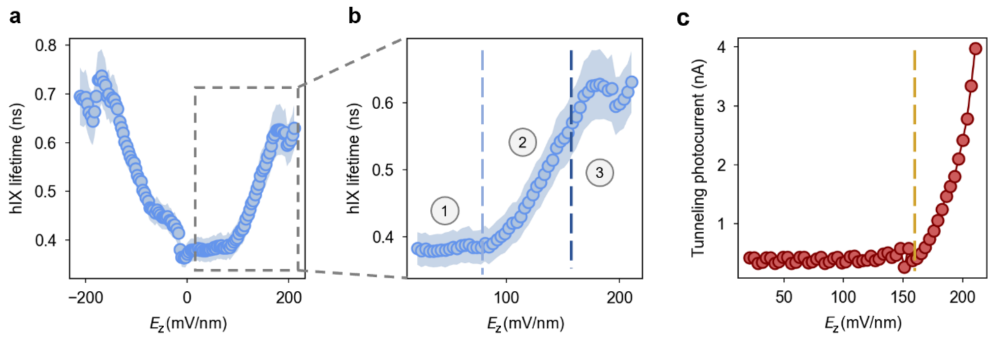

Figure S8. a, Field-dependent lifetime measurements of device B, as reported in Figure 3a in the main text. Here, we focus on the region $E_z > 20\,\mathrm{mV/nm}$ (dashed grey rectangle). **b,** For a positive electric field, three main regions are highlighted. Regions 1 ($20\,\mathrm{mV/nm} < E_z < 80\,\mathrm{mV/nm}$) and 2 ($80\,\mathrm{mV/nm} < E_z < 160\,\mathrm{mV/nm}$) are characterized by a monotonic increase in τ_{tot} and correspond to regions of dominating $K\Lambda$ and $K\Lambda'$ transitions, respectively. Instead, region 3 ($E_z > 160\,\mathrm{mV/nm}$) exhibits a sudden drop in τ_{tot}, which is attributed to the appearance of a nonradiative recombination channel given by the dissociation of hIXs. **c,** Tunneling photocurrent measured in device B. The rise in signal observed in region 3 is related to the dissociation of hIX at high fields, allowing the extraction of carriers tunneling from WSe_2 to the top and bottom gate electrodes through the hBN layers. Lifetime data are presented as mean values (dots) and corresponding standard deviations (shaded surrounding area).

8. Experimental setup and optical measurements

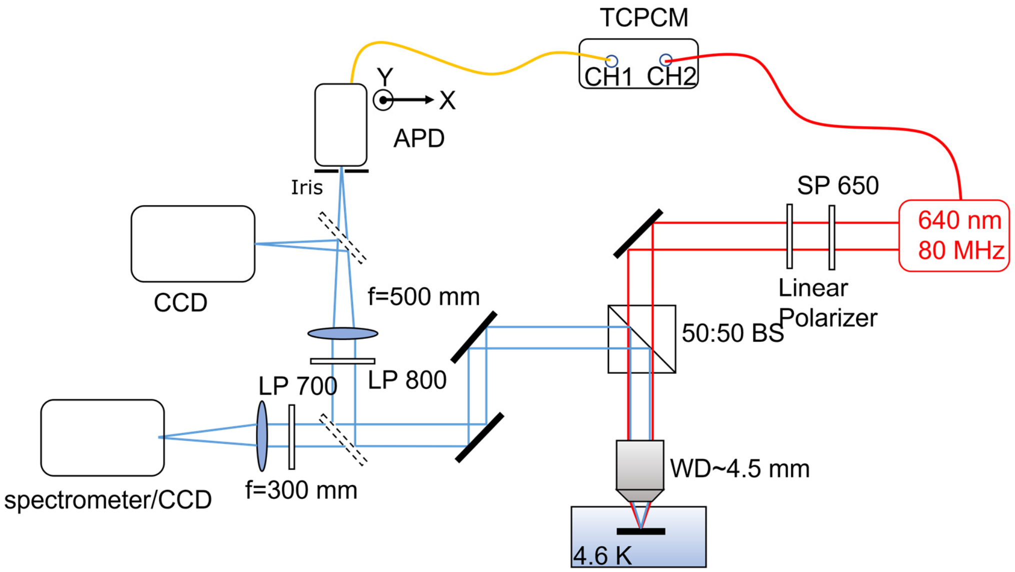

Figure S9. Schematic representation of the complete optical setup for spectroscopic measurements, CCD imaging and scanning APD spatiotemporally-resolved measurements.

All optical measurements were performed in vacuum in a He-flow cryostat at 4.6 K, unless stated otherwise. A schematic representation of the employed optical setup is shown in Figure S9. Hybrid excitons are optically generated in our sample by employing a picosecond pulsed solid state laser at a repetition rate of 80 MHz in time resolved measurements, while the same laser is driven in continuous wave mode for steady state measurements. The incident laser is linearly polarized. A short-pass 650 nm (SP650) filter is placed at the excitation arm, in order to filter out 2nd order components of the laser light. A confocal microscope is used to optically excite hIXs and to collect the emitted photons through the same objective, with a working distance (WD) of ~ 4.5 mm and NA = 0.65. The light emitted from the sample is separated from the laser excitation by a 50:50 (reflection:transmission) beam splitter (BS). A long-pass 700 nm (LP700) filter is used for spectral measurements, while for real-space exciton cloud imaging a long-pass 800 nm (LP800) filter is placed in front of the CCD camera or the APD sensor. Motorized flip mirrors are used to send the collected photons to either a spectrometer (Princeton Instruments), to the CCD sensor or to our homemade scanning APD system. The spectrometer is used to measure the PL spectrum, the CCD camera to image the steady-state hIX cloud expansion, and the scanning APD system is employed for spatiotemporally resolved hIX transport measurements.

The APD sensor (Excelitas Technologies, SPCM-AQRH-16) systematically moves within the focal plane, as it is mounted on a two-dimensional (2D) motorized translational stage. The output signal of the APD is connected to a time-correlated photon counting module (TCPCM) with a resolution of 12 ps r.m.s. (PicoQuant, PicoHarp 300), which measures the arrival time of each photon. For the measurements in this work, we set the time bin to 16 ps to record photon clicks. The single photon timing resolution of the APD is about ~ 350 ps, which is the main time resolution-limiting factor for the whole setup. Its high quantum efficiency at the near-infrared range (~ 55% at 850 nm), however, combined with its low dark count rate (~ 20 Hz) enables us to measure the spatial profile of the long-lived but rather weak emission of hIXs with a good signal to noise ratio (SNR > 10). The magnification of the scanning APD imaging system is about 150. In the measurements of 2D time-resolved PL images (Figure 4), the two motors are scanned with a step size of 20 μm, which corresponds to ~ 130 nm on the sample. Figure S9 shows images of the excitation laser acquired by the CCD camera and the scanning APD system. The area of the excitation laser is extracted and plotted in Figure S10a as a function of time.

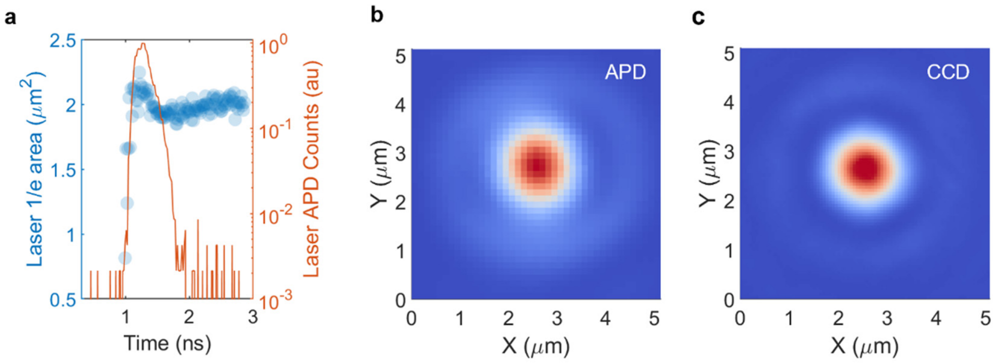

Figure S10. a, On the left axis the laser area expansion in time measured from the scanning APD system. A constant laser area of approximately $2\mu m^2$ is observed within the time range of interest. On the right axis the time resolved laser profile is shown. **b,** Spatial reconstruction of the focused laser image measured from the scanning APD system. **c,** Focused laser image obtained from the CCD camera (Andor Ixon), consistent with that shown in (b).

9. Drift-diffusion equation and transport simulations

To gain microscopic access to the spatiotemporal dynamics of hybrid excitons, we solve the microscopically derived two-dimensional drift-diffusion equation[3,29,30]:

$$\partial_t n(\mathbf{r}, \mathrm{t}) = D\nabla^2 n(\boldsymbol{r}, t) + \mu_m \nabla \cdot \left(\nabla\left(\Delta E\big(n(\boldsymbol{r}, t)\big)\right) n(\boldsymbol{r}, t)\right) - \frac{n(\boldsymbol{r}, t)}{\tau} \tag{14}$$

where D is the diffusion coefficient, $\mu_m = \frac{D}{k_B T}$ is the exciton mobility, $\Delta E\big(n(\boldsymbol{r}, t)\big)$ is the density-dependent energy renormalization and τ is the exciton lifetime. We can further expand the energy renormalization term by introducing an electric-field-dependent factor $C(E_z)$:

$$\begin{aligned} \partial_t n(\mathbf{r}, \mathrm{t}) &= D\nabla^2 n(\boldsymbol{r}, t) + \mu_m \nabla \cdot \left(\nabla\big(C(\boldsymbol{r}, t, E_z) \cdot n(\boldsymbol{r}, t)\big) n(\boldsymbol{r}, t)\right) - \frac{n(\boldsymbol{r}, t)}{\tau} = \\ &= D\nabla^2 n(\boldsymbol{r}, t) + \mu_m\, C(\boldsymbol{r}, t, E_z)\nabla^2 n(\boldsymbol{r}, t) - \frac{n(\boldsymbol{r}, t)}{\tau} = \\ &= D_{\mathrm{eff}}(\boldsymbol{r}, t, E_z)\nabla^2 n(\boldsymbol{r}, t) - \frac{n(\boldsymbol{r}, t)}{\tau} \end{aligned} \tag{15}$$

with $D_{\mathrm{eff}}(r, t, \boldsymbol{E_z}) = D + \mu_m\, C(r, t, E_z)$. We initialize the distribution as a Gaussian profile:

$$n(x, y, 0) = n_0 \exp\left(-\frac{(x^2 + y^2)}{\sigma_0^2}\right), \tag{16}$$

with the initial spot size adapted to the laser spot size (Supplementary Note 8). To provide an initial exciton density for transport simulations, we can follow our reasoning in Supplementary Note 6. Since we are analyzing species at equal and opposite electric fields ($E_z = \pm 300$ mV/nm), it is reasonable to assume that the generation factors for both species are comparable. In particular, since $n_{hd}/n_{ld} = G_{hd}\tau_{hd}/G_{ld}\tau_{ld}$ and $\tau_{hd}/\tau_{ld} \simeq 1.67$, we conclude that the exciton densities for both species must lie in the same range. Thus, we use an initial density of $n_0 \sim 10^{12}$ cm^{-2} for both low and high-d species based on a best-fit approach to our experimental time-resolved transport at long times, in the regime of classical diffusion. The obtained results in the regime of anomalous diffusion are further discussed in the main text.

The drift-diffusion equation in Eq. (15) is solved with a Runge-Kutta method giving access to the spatiotemporal dynamics of the hybrid exciton density. Given $n(r,\ t)$, we define the hIX area $A(t) = \pi x_0^2(t)$ where $x_0^2(t)$ is extracted from the condition $\frac{n(x_0, 0, t)}{max\left(n(x_0, 0, t)\right)} = \frac{1}{e}$ for fixed times t. Furthermore, we extract the effective diffusivity $D_{\mathrm{eff}}(t) \equiv \frac{1}{4\pi}\frac{dA}{dt}$, which coincides with the chosen low-density diffusivity at infinity, $D_{\mathrm{eff}}(\infty) = D$. The low-density diffusion coefficient $D = 0.322\ \mathrm{cm}^2/s$ is obtained from experimental measurements (Supplementary Note 10). The exciton lifetimes at high positive and negative fields are extracted from the experimental data as $\tau_{\mathrm{hd}} = \tau(E_z = 300\ \mathrm{mV/nm}) = 680\ \mathrm{ps}$ and $\tau_{\mathrm{ld}} = \tau(E_z = -300\ \mathrm{mV/nm}) = 375\ \mathrm{ps}$ (Supplementary Note 7).

10. Estimation of the conventional diffusivity of hIXs

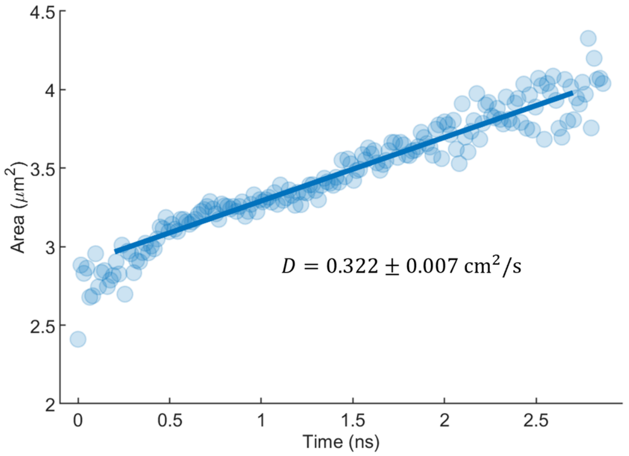

Figure S11. Effective hIX cloud area expansion in time for $E_z = 0$ mV/nm. From a linear fit to the effective area expansion we obtain $D = 0.322 \pm 0.007$ cm^2/s.

Excitons in WSe_2 homobilayers at vanishing applied electric fields are predominantly intralayer in nature, with dipoles that are randomly oriented (Supplementary Note 2). Thus, Coulomb repulsions are inhibited by the lack of alignment between dipoles, and excitons are expected to diffuse conventionally. In Figure S11, we show the measured effective hIX cloud expansion at $E_z = 0$ mV/nm, characterized by a linear trend in time. Thus, a fixed value of conventional diffusivity is extracted from linearly fitting the measured data, obtaining $D = \frac{1}{4\pi}\frac{dArea}{dt} \simeq 0.32\ \mathrm{cm^{-2}s^{-1}}$. This result is comparable with the slope of the effective area expansion in the case of high electric fields for $t \to \infty$ (Figure 4d-e). In fact, all hIXs exhibit a diffusivity that settles to a regime of conventional diffusive transport as $D_{\mathrm{eff}}^{hd}(\infty) = D_{\mathrm{eff}}^{ld}(\infty) = D$. The measured diffusivity factor is employed as an experimental parameter in the transport simulations of hIXs with different levels of hybridization (Supplementary Note 9), allowing to obtain a good estimate of the $\lim_{t\to 0^+} D_{\mathrm{eff}}^{hd}$ and $\lim_{t\to 0^+} D_{\mathrm{eff}}^{ld}$, which otherwise would not be possible due to the finite and limited time resolution of the instrumental setup.

REFERENCES

1. Purdie, D. G. et al. Cleaning interfaces in layered materials heterostructures. Nat. Commun. 9, 5387 (2018).
2. Ovesen, S. et al. Interlayer exciton dynamics in van der Waals heterostructures. Commun. Phys. 2, 1–8 (2019).
3. Erkensten, D., Brem, S., Perea-Causín, R. & Malic, E. Microscopic origin of anomalous interlayer exciton transport in van der Waals heterostructures. Phys. Rev. Mater. 6, 094006 (2022).
4. Hagel, J., Brem, S., Linderälv, C., Erhart, P. & Malic, E. Exciton landscape in van der Waals heterostructures. Phys. Rev. Res. 3, 043217 (2021).
5. Brem, S. et al. Hybridized intervalley moiré excitons and flat bands in twisted WSe_2 bilayers. Nanoscale 12, 11088–11094 (2020).
6. Hagel, J., Brem, S. & Malic, E. Electrical tuning of moiré excitons in $MoSe_2$ bilayers. Preprint at http://arxiv.org/abs/2207.01890 (2022).
7. Lindlau, J. et al. The role of momentum-dark excitons in the elementary optical response of bilayer WSe_2. Nat. Commun. 9, 2586 (2018).
8. Deilmann, T. & Thygesen, K. S. Finite-momentum exciton landscape in mono- and bilayer transition metal dichalcogenides. 2D Mater. 6, 035003 (2019).
9. Jin, Z., Li, X., Mullen, J. T. & Kim, K. W. Intrinsic Transport Properties of Electrons and Holes in Monolayer Transition Metal Dichalcogenides. Phys. Rev. B 90, 045422 (2014).
10. Efimkin, D. K. & MacDonald, A. H. Many-body theory of trion absorption features in two-dimensional semiconductors. Phys. Rev. B 95, 035417 (2017).
11. Laturia, A., Van de Put, M. L. & Vandenberghe, W. G. Dielectric properties of hexagonal boron nitride and transition metal dichalcogenides: from monolayer to bulk. Npj 2D Mater. Appl. 2, 1–7 (2018).
12. Lu, X. & Yang, L. Stark effect of doped two-dimensional transition metal dichalcogenides. Appl. Phys. Lett. 111, 193104 (2017).
13. Wang, Z., Chiu, Y.-H., Honz, K., Mak, K. F. & Shan, J. Electrical Tuning of Interlayer Exciton Gases in WSe_2 Bilayers. Nano Lett. 18, 137–143 (2018).
14. Erkensten, D., Brem, S. & Malic, E. Exciton-exciton interaction in transition metal dichalcogenide monolayers and van der Waals heterostructures. Phys. Rev. B 103, 045426 (2021).
15. Shahnazaryan, V., Iorsh, I., Shelykh, I. A. & Kyriienko, O. Exciton-exciton interaction in transition-metal dichalcogenide monolayers. Phys. Rev. B 96, 115409 (2017).
16. Rivera, P. et al. Valley-polarized exciton dynamics in a 2D semiconductor heterostructure. Science 351, 688–691 (2016).
17. Observation of Exciton Redshift–Blueshift Crossover in Monolayer WS_2 . Nano Letters. https://pubs.acs.org/doi/10.1021/acs.nanolett.7b01034.
18. Ciarrocchi, A. et al. Polarization switching and electrical control of interlayer excitons in two-dimensional van der Waals heterostructures. Nat. Photonics 13, 131–136 (2019).
19. Unuchek*, D. et al. Valley-polarized exciton currents in a van der Waals heterostructure. Nat. Nanotechnol. 1–6 (2019).
20. Sun, Z. et al. Excitonic transport driven by repulsive dipolar interaction in a van der Waals heterostructure. Nat. Photonics 16, 79–85 (2022).
21. Negoita, V., Snoke, D. W. & Eberl, K. Huge density-dependent blueshift of indirect excitons in biased coupled quantum wells. Phys. Rev. B 61, 2779–2783 (2000).
22. Jauregui, L. A. et al. Electrical control of interlayer exciton dynamics in atomically thin heterostructures. Science 366, 870–875 (2019).
23. Lien, D.-H. et al. Electrical suppression of all nonradiative recombination pathways in monolayer semiconductors. Science 364, 468–471 (2019).
24. Linardy, E. et al. Harnessing Exciton–Exciton Annihilation in Two-Dimensional Semiconductors. Nano Lett. 20, 1647–1653 (2020).
25. Chow, C. M. E. et al. Monolayer Semiconductor Auger Detector. Nano Lett. 20, 5538–5543 (2020).
26. Sushko, A. et al. Asymmetric photoelectric effect: Auger-assisted hot hole photocurrents in transition metal dichalcogenides. Nanophotonics 10, 105–113 (2021).
27. Kash, J. A., Mendez, E. E. & Morkoç, H. Electric field induced decrease of photoluminescence lifetime in GaAs quantum wells. Appl. Phys. Lett. 46, 173–175 (1985).
28. Sivalertporn, K., Mouchliadis, L., Ivanov, A. L., Philp, R. & Muljarov, E. A. Direct and indirect excitons in semiconductor coupled quantum wells in an applied electric field. Phys. Rev. B 85, 045207 (2012).
29. Ivanov, A. L. Quantum diffusion of dipole-oriented indirect excitons in coupled quantum wells. EPL Europhys. Lett. 59, 586 (2002).
30. Rosati, R. et al. Dark exciton anti-funneling in atomically thin semiconductors. Nat. Commun. 12, 7221 (2021).
